# The Low-Cost Compound Lignosulfonic Acid (LA) Exhibits Broad-Spectrum Anti-HIV and Anti-HSV Activity and Has Potential for Microbicidal Applications

**DOI:** 10.1371/journal.pone.0131219

**Published:** 2015-07-01

**Authors:** Stephanie C. Gordts, Geoffrey Férir, Thomas D’huys, Mariya I. Petrova, Sarah Lebeer, Robert Snoeck, Graciela Andrei, Dominique Schols

**Affiliations:** 1 Rega Institute for Medical Research, University of Leuven, Leuven, Belgium; 2 Centre of Microbial and Plant Genetics, University of Leuven, Leuven, Belgium; 3 Department of Bioscience Engineering, Antwerp University, Antwerp, Belgium; University Hospital Zurich, SWITZERLAND

## Abstract

**Objectives:**

Lignosulfonic acid (LA), a low-cost lignin-derived polyanionic macromolecule, was extensively studied for its anti-HIV and anti-HSV activity in various cellular assays, its mechanism of viral inhibition and safety profile as potential microbicide.

**Results:**

LA demonstrated potent inhibitory activity of HIV replication against a wide range of R5 and X4 HIV strains and prevented the uptake of HIV by bystander CD4^+^ T cells from persistently infected T cells *in vitro* (IC_50_: 0.07 – 0.34 μM).

LA also inhibited HSV-2 replication *in vitro* in different cell types (IC_50_: 0.42 – 1.1 μM) and in rodents *in vivo*. Furthermore, LA neutralized the HIV-1 and HSV-2 DC-SIGN-mediated viral transfer to CD4^+^ T cells (IC_50_: ∼1 μM). In addition, dual HIV-1/HSV-2 infection in T cells was potently blocked by LA (IC_50_: 0.71 μM). No antiviral activity was observed against the non-enveloped viruses Coxsackie type B4 and Reovirus type 1.

LA is defined as a HIV entry inhibitor since it interfered with gp120 binding to the cell surface of T cells. Pretreatment of PBMCs with LA neither increased expression levels of cellular activation markers (CD69, CD25 and HLA-DR), nor enhanced HIV-1 replication. Furthermore, we found that LA had non-antagonistic effects with acyclovir, PRO2000 or LabyA1 (combination index (CI): 0.46 – 1.03) in its anti-HSV-2 activity and synergized with tenofovir (CI: 0.59) in its anti-HIV-1 activity. To identify mechanisms of LA resistance, we generated *in vitro* a mutant HIV-1 NL4.3^LAresistant^ virus, which acquired seven mutations in the HIV-1 envelope glycoproteins: S160N, V170N, Q280H and R389T in gp120 and K77Q, N113D and H132Y in gp41. Additionally, HIV-1 NL4.3^LAresistant^ virus showed cross-resistance with feglymycin, enfuvirtide, PRO2000 and mAb b12, four well-described HIV binding/fusion inhibitors. Importantly, LA did not affect the growth of vaginal *Lactobacilli* strains.

**Conclusion:**

Overall, these data highlight LA as a potential and unique low-cost microbicide displaying broad anti-HIV and anti-HSV activity.

## Introduction

According to UNAIDS’ latest results, about 2.1 million new human immunodeficiency virus (HIV) infections still occurred worldwide in 2013 [1]. Multiple studies indicate the importance of the interaction between genital herpes simplex type 2 (HSV-2) infections and HIV-1 on the sexual transmission in women [2–6]. The association of HSV-2 with significantly higher amounts of HIV-1 in plasma and genital secretions suggests that antiviral treatment of solely HSV-2 with nucleoside analogues (e.g. acyclovir) could result in a reduced replication rate of HIV-1. Although condom use is still the best way to protect men and women against sexually transmitted pathogens such as HIV and HSV-2, it would be of great benefit for women to develop self-administrating topical microbicides (e.g. vaginal/rectal gels, intravaginal ring systems, suppositories, pills) containing one or more antiviral agents with an exquisite activity against both HSV-2 and HIV-1.

At present, the HIV-1 nucleotide reverse transcriptase inhibitor (NtRTI) tenofovir (Viread) is the most promising microbicidal compound evaluated in clinical trials so far [7]. Topically applied gel-formulated tenofovir has been shown to reduce the sexual transmission of HIV-1 significantly by 39% overall and surprisingly also of HSV-2 by 51% [8]. However, the observed inhibitory activities of tenofovir on HSV-2 replication by targeting the viral DNA polymerase was only achieved at higher drug levels [9].

Acyclovir (Zovirax) is the gold standard drug for treatment of HSV infections and belongs to a group of synthetic drugs called nucleoside analogs [10]. The compound specifically inhibits the herpes DNA polymerase and has little effect on the host cell DNA polymerase. However, studies proved that long-term administration of acyclovir in immunocompromised patients could result in drug-resistant HSV strains [11]. Lisco *et al*. [12] demonstrated that acyclovir can even act as an HIV-1 reverse transcriptase inhibitor in herpes virus-infected cells, while McMahon *et al*. [13] found a consistent inhibition of HIV-1 in the absence of herpes viruses. The observed activity of tenofovir and the inconsistent findings of acyclovir indicate the need for antiviral agents targeting both viruses with equal potency. Recent insights highlight also the emergence of acyclovir-resistant HSV strains in immunocompetent individuals treated for herpetic keratitis or encephalitis [11].

Entry inhibitors may even have a better profile as potential microbicide candidates as they prevent infection of the target cells already in the (vaginal/rectal) lumen. Therefore, we focus on the low-cost molecule lignosulfonic acid (LA), which belongs to the family of lignin-derived macromolecules, byproducts formed during the conversion of woodpulp into paper [14]. Previously published reports demonstrated that LA has some very interesting biological properties such as long time usage as an animal feed additive due to anti-pepsin activity and protective effects against gastric ulcer development [15, 16]. Preliminary activity of different water-soluble lignins against certain HIV-1 isolates was reported previously [17, 18].

Here, we report an extensive evaluation of the consistent broad-spectrum anti-HIV and anti-HSV activity of a low molecular weight variant of LA (mw: ∼8000 g/mol) in various HIV and HSV target cell lines and *in vivo* in a mouse model. We also demonstrate its excellent safety profile at the cellular level and at the level of vaginal *Lactobacilli* microbiota. Hereby highlighting its potential use for topical microbicidal applications.

## Materials and Methods

### Cell lines and virus strains

The CD4^+^ T-lymphoma cell lines C8166, SupT1 and HUT-78 were obtained from the American Type Culture Collection (ATCC, Manassas, VA, USA). The MT-4 cells were a gift from Dr. L. Montagnier (formerly at the Pasteur Institute, Paris, France)[19]. Persistently HIV-1 IIIB HUT-78 (HUT-78/IIIB) cells were generated as described earlier [20]. The B-lymphoma cell line Raji.DC-SIGN^+^ was obtained from Dr. L. Burleigh (Pasteur Institute)[21]. All cells were cultured in RPMI-1640 medium (Invitrogen, Merelbeke, Belgium) containing 10% FCS (Hyclone, Perbio Science, Aalst, Belgium) and 1% l-glutamine (Invitrogen).

The embryonic HEK293T cells were ordered from the ATCC. TZM-bl cells [22, 23] were a kind gift from Dr. G. Vanham (ITG, Antwerp, Belgium). Both cell lines were cultured in DMEM supplemented with 10% FCS and 1% HEPES (Invitrogen).

Peripheral blood mononuclear cells (PBMCs) were isolated out of buffy coats from healthy donors, derived from the blood transfusion center (Red Cross, Belgium) by density centrifugation. The cells were then cultured in RPMI-1640 medium supplemented with 10% FCS and 1% l-glutamine. PBMCs were stimulated with 2 μg/ml phytohemagglutinin (PHA) for 3 days at 37°C, before further usage in HIV infection assays. The preparation of monocyte/macrophages (MDM) was described previously [24].

Monocyte-derived dendritic cells (MDDCs) were generated from freshly isolated PBMCs (cfr. *supra*). PBMCs were gently rotated at 4°C to form monocyte aggregates. After sedimentation of the mononuclear cell aggregates, the pellet was resuspended in RPMI medium and incubated for 90 min. at 5% CO_2_, 37°C to allow for monocyte adherence. After the incubation period, non-adherent cells were carefully removed by washing three times with PBS. Adherent cells were cultured in RPMI culture medium supplemented with 25 ng/ml IL-4 and 50 ng/ml GM-CSF (Peprotech, London, United Kingdom). Four days later, culture medium was changed with fresh medium containing the same cytokine composition. After 6 days, IL-4 and GM-CSF had induced differentiation of monocytes into immature MDDCs as shown by phenotypical analysis by fluorescently labeled anti-CD1a, anti-CD14, anti-CD80, anti-CD86, anti-DC-SIGN and anti-HLA-DR antibodies (all from BD Biosciences, Erembodegem, Belgium) (S1 Fig).

The human embryonic lung fibroblasts (HEL cells) were obtained from the ATCC and cultured in MEM containing 10% FCS, 1% l-glutamine and 0.3% sodium bicarbonate (Invitrogen).

The HIV-1 strains NL4.3 (X4), IIIB (X4) and BaL (R5) were obtained from the AIDS Research and Reference Reagent Program (Division of AIDS, NIAID, NIH). HIV-1 strain HE (X4/R5) was originally isolated from a Belgian AIDS patient [25]. The HIV-2 strain ROD was obtained from the Medical Research Council (MRC, London, UK). The characterization of the *in vitro* generated HIV-1 NL4.3 and IIIB strains resistant to the entry inhibitors 2G12 monoclonal antibody (mAb), AMD3100, enfuvirtide (T20), feglymycin (FGM) and PRO2000 were described previously [24, 26, 27].

The HIV-1 clinical isolates (UG273, US2, BK132, ETH2220, DJ259, SE365, BZ163, BCF-DIOUM, HH8793, BCF-KITA, BCF-06), representing different subtypes were kindly provided by Dr. J.L. Lathey (formerly at BBI Biotech Research Laboratories, Gaithersburg, MD, USA). Their HIV coreceptor usage was determined in our lab using U87.CD4.CXCR4 and U87.CD4.CCR5-transfected cells, as described previously [28].

The HSV-1 KOS and HSV-2 G were used as reference laboratory HSV strains. The HSV-1 wild-type (RV-174), HSV-1 thymidine kinase-deficient (TK^-^) (RV-179, RV-117), HSV-2 wild-type (RV-124, RV-24) and HSV-2 TK^-^ (RV-129, BA19026589) clinical isolates were derived from virus-infected individuals in Belgium. They were obtained as part of a translational research program granted by the Belgian Ministry of Health as part of the National Cancer Plan for the diagnosis of drug resistance in herpesviruses. The viruses were obtained and used as approved according to the rules of Belgian equivalent of IRB (Institutional review board) (Departement Leefmilieu, Natuur en Energie, protocol SBB219 2011/0011, and the Biosafety Committee at the University of Leuven).

### Test compounds

The polyanionic compounds LA (mw: ∼8000 g/mol) and dextran sulfate (mw: ∼5000 g/mol) were purchased from Sigma-Aldrich (Diegem, Belgium). PRO2000 (mw: ∼5000 g/mol) was kindly provided by Dr. A.T. Profy (Formerly at Indevus Pharmaceuticals Inc., Lexington, MA, USA). *Hippeastrum hybrid* agglutinin (HHA; mw: 50 kDa) was obtained from EY Labs (San Mateo, CA, USA). Griffithsin (GRFT; mw: 25.4 kDa) was kindly provided by Dr. K.E. Palmer (University of Louisville, KY, USA). The bacterial antibiotic peptides feglymycin (FGM; mw: 1.9 kDa) and Labyrintopeptin A1 (LabyA1; mw: 2.1 kDa) were provided by Dr. R.D. Süssmuth (Technische Universität Berlin, Germany). Acyclovir (mw: 225 g/mol) was obtained from GlaxoSmithKline (Brentford, UK). Maraviroc (mw: 514 g/mol) and AMD3100 (mw: 830 g/mol) were donated by Dr. G. Bridger (at that time at AnorMed, Langley, Canada). Enfuvirtide (T20; mw: 4.5 kDa) was provided by Dr. E. Van Wijngaerden (University Hospitals, Leuven, Belgium). The mitogenic lectin PHA (mw: 128 kDa) was ordered from Sigma-Aldrich. Tenofovir (mw: 287.21 g/mol) was obtained from Gilead Sciences (Foster City, CA, USA). The mAb b12 was ordered from Polymun Scientific (Vienna, Austria).

### HIV-1 replication assays in MT-4-, TZM-bl- and primary cells

The anti-HIV assays in MT-4 cells and PBMCs have been described previously [29]. MT-4 cells (1x10^6^ cells/ml; 50 μl) were pre-incubated for 30 min. at 37°C with the test compounds in 96-well plates (Falcon, BD Biosciences). The laboratory HIV strains (HE, NL4.3, IIIB and ROD) were added according to the 50% tissue culture infectious dose (TCID_50_) of the viral stock. Cytopathicity was scored microscopically in MT-4 cells 5 days post-infection and IC_50_s were calculated spectrophotometrically using MTS/PES. For the latter viability assay, the ‘CellTiter96 Aqueous One Solution Proliferation Assay’ (Promega, Leiden, The Netherlands) kit was used.

The firefly luciferase and *Escherichia coli* β-galactosidase expressing CD4^+^, CXCR4^+^, CCR5^+^ TZM-bl cells (50 μl; 2x10^5^ cells/ml) were resuspended in cell culture medium supplemented with 15 μg/ml diethylaminoethyl-dextran (DEAE-Dextran; Sigma-Aldrich, Diegem, Belgium) and pre-incubated for 30 min. at 37°C in 96-well plates with in cell culture medium diluted test compounds (100 μl). Next, the laboratory HIV-1 strain R5 BaL was added (50 μl) according to the TCID_50_ of the viral stock. Two days post-infection, viral replication was measured by luminescence. Steadylite plus reagent (Perkin Elmer, Zaventem, Belgium) was mixed with lyophilized substrate according to manufacturer’s guidelines. Supernatant (120 μl) was removed and 75 μl Steadylite plus substrate solution was added to the 96-well plates. Next, the plates were incubated in dark for 10 min. in a closed plate shaker (PHMP, Grant, Shepreth, Cambridgeshire, UK). Finally, cell lysis was scored microscopically and 100 μl was transferred to white lumitrac 96-well plates (Greiner Bio-One, Frickenhausen, Germany) to measure the relative luminescence units (RLUs) using the SpectraMax L microplate reader and Softmax Pro software (Molecular Devices, Sunnyvale, CA, USA) with an integration time of 0.6 sec. and dark adapt of 5 min.

Freshly isolated PBMCs were stimulated with 2 μg/ml PHA for 3 days at 37°C before further use in anti-HIV assays. These PHA-stimulated PBMCs (200 μl; 5x10^5^ cells/ml) were seeded in a 48-well plate (Costar, Elscolab NV, Kruibeke, Belgium) and pre-incubated for 30 min. at 37°C with various concentrations of LA (250 μl) in the presence of 2 ng/ml interleukin (IL)-2 (Roche Applied Science, Vilvoorde, Belgium). Next, (500 μl) 1500 pg/well of HIV-1 BaL and 500 pg/well of HIV-1 clinical isolates was given and at day 3 and 6 of infection, 2 ng/ml of IL-2 was added again. Finally, 10 days post-infection, supernatant was collected for HIV-1 p24 Ag ELISA (Perkin Elmer) according to manufacturer’s guidelines.

MDM were seeded in 48-well plates in 1 ml medium. After removal of 800 μl of medium, 250 μl of various concentrations of LA was given in triplicate. After 30 min. at 37°C, 1000 pg/well of HIV-1 BaL was added. Three weeks post-infection, supernatant was collected and viral replication measured using HIV-1 p24 Ag ELISA.

### HIV-1 time-of-drug addition experiments

The methodology of time-of-drug addition studies was described previously [30]. Briefly, MT-4 cells (1x10^6^ cells/ml) were infected with HIV-1 NL4.3. The test compounds were added in a range from 0–5 h post-infection. Afterwards, HIV-1 replication was determined by p24 HIV-1 Ag ELISA.

### Virus-inactivation assay in MT-4 cells

Fifty microliter of product solution (LA low, PRO 2000, HHA, aldrithiol) at a starting concentration of 100 μg/ml was incubated with 50 μl of HIV-1 NL4.3 virus stock (2,000,000 pg/ml) in Nunc tubes (Sigma Aldrich). After 1 hour of incubation at room temperature, all virus samples (with or without product) were diluted to 2,000 pg/ml, 1,000 pg/ml and 200 pg/ml. Subsequently, 100 μl aliquots of the dilutions were added to MT-4 cells (100 μl, 50,000 cells/well) in a 96-well plate. Experiments were carried out in quintuplicate. The MT-4 cells were exposed to concentrations (dilutions) of product that were well below their IC_50_ values. Degree of infection was assessed with the cellular viability MTS/PES assay after 5 days of incubation. IC_50_ values were determined based on the reference values obtained with the untreated positive control.

### Anti-HSV replication assays in epithelial HEL cells

The antiviral assays of HSV replication in HEL cells was described earlier [31]. Shortly, confluent cell cultures in 96-well plates were incubated with various concentrations of LA and simultaneously infected with 100 TCID_50_ of laboratory HSV strains or clinical isolates for 3 days at 37°C before viral cytopathogenic effect (CPE) was measured microscopically. The anti-HSV activity is expressed as the IC_50_ (concentration required to reduce virus induced CPE by 50%).

### HSV-2 time-of-drug addition studies

The time-of-drug addition experiments with HSV-2 G were performed identically as the viral replication assays in HEL cells, but the compounds were added separately at the time of infection (0 h) and 2 h post-infection at 1 fixed concentration: LabyA1 9.6 μM; LA 0.5 μM and acyclovir 10 μM. After 3 days, HSV-2 induced CPE was scored microscopically.

### MDDCs HSV-2 infection assays

MDDCs were seeded in 48-well plates (12x10^5^ cells/200 μl/well) and pre-incubated with various concentrations of LA (250 μl/well) for 30 min. at 5% CO_2_, 37°C. After the incubation period, 50 μl of 1/40 diluted HSV-2 G was added to the wells, whether or not in the presence of compound. After 5 days of incubation, cells were harvested and processed for further flow cytometric side-scatter analysis. Viral titers of the samples were determined in human embryonic lung fibroblasts [9].

### HSV-2 replication assays in HIV-1 susceptible CD4^+^ T-lymphoma cells

The HIV-1 susceptible T-lymphoma cell lines MT-4 and C8166 (50 μl; 5x10^4^ cells/well) were seeded in 96-well plates and pre-incubated with various concentrations of LA (100 μl). After 30 min. at 37°C, 50 μl of 1/30-diluted HSV-2 G was added. Cytopathicity was analyzed after 3 days in MT-4 cells and after 4 days in C8166 T cells. The IC_50_s were calculated using MTS/PES as described above. In the dual HIV-1/HSV-2 infection conditions, HSV-2 replication was measured with flow cytometry using the mouse mAb anti glycoprotein B (agB.10.B.7.) (Abcam, UK).

### Gp120 virus binding assay

CD4^+^ SupT1 T cells (200 μl; 2.5x10^6^ cells/ml) were mixed with 200 μl of medium/compounds and high amounts of HIV-1 NL4.3 (100 μl; p24 of 2x10^6^ pg/ml) for 2h on room temperature in a conical 15 ml polypropylene tube (Falcon, BD). After 2 washing steps with PBS containing 2% FCS (PBS/FCS2%), virus binding was detected using 1/50-diluted anti-gp120 mAb NEA-9205 (200 μl; NEN, Boston, MA, USA) or the anti-gp120 2G12 mAb (Polymun Scientific GmbH, Austria). After an incubation of 30 min. at 4°C and 2 wash steps, 200 μl of 1/100 diluted PE-conjugated Goat-anti-Mouse (GaM-PE; Biolegend, ImTec Diagnostics NV, Antwerp, Belgium) or 1/50 diluted FITC-conjugated polyclonal Rabbit-anti-Human IgG/FITC (RaH-IgG-FITC; Dako, Heverlee, Belgium) was added and incubated at the same conditions. After washing, the samples were fixed with 300 μl of a 1% paraformaldehyde solution and analyzed by flow cytometry (FACSCalibur; BD). Aspecific binding was determined using GaM-PE only.

CD4^+^ SupT1 T cells (200 μl; 2.5x10^6^ cells/ml) were mixed with 200 μl medium or compounds for 15 min. at room temperature in a conical 15 ml polypropylene tube. After 2 washing steps with PBS/FCS2%, the cells were incubated with high amounts of HIV-1 NL4.3 (100 μl; p24 of 2x10^6^ pg/ml) for 2 h again on room temperature. After extensive washing, virus binding was detected using the anti-gp120 mAb NEA-9205 (1/50-fold dilution in PBS/FCS2%) and 1/100 diluted PE-conjugated Goat-anti-Mouse (GaM-PE) at incubation conditions as described above. Finally, the samples were fixed with a 1% paraformaldehyde solution and analyzed by flow cytometry.

### HIV-1 cell-cell cocultivation assay

This method was described in detail previously [20]. In brief, various concentrations of LA (100 μl) were added in a 96-well plate along with CD4^+^ SupT1 T cells (1x10^5^ cells/50 μl). The persistently HIV-1 IIIB infected HUT-78 cells (HUT-78/IIIB) were washed to remove free viral particles, resuspended at a density of 1x10^5^ cells/50 μl and immediately mixed with the SupT1 T cells for 24 h at 37°C. The next day, giant cell formation was scored microscopically and afterwards, flow cytometry was used to calculate the IC_50_s. The target SupT1 T cell population was stained with PE-conjugated anti-CD28 (CD28-PE, BD) diluted in PBS/FCS2% for 30 min. at 4°C. After two wash steps, the cells were fixed with a 1% paraformaldehyde solution. Acquisition and analysis occurred on a FACSArray flow cytometer (BD) using Windows-based FACSArray System. Aspecific binding was determined using SimulTest control (IgGγ1-FITC/IgGγ2a-PE; BD).

### HIV-1/DC-SIGN capture assay

The capture assay was performed in conical polypropylene 15 ml tubes. LA, PRO2000 and HHA (200 μl) were diluted in medium and pre-incubated with high amounts of 100 μl of HIV-1 HE (R5/X4; p24 of ∼2.5x10^6^ pg/ml) for 30 min. at 37°C before 200 μl of Raji.DC-SIGN^+^ cells (5x10^6^ cells/ml) were added. After 1 h of incubation at 37°C, the cells were extensively washed with medium and resuspended in 200 μl of freshly prepared triton buffer (Triton-X100/PBS solution). Samples were stored at -20°C till HIV-1 p24 Ag ELISA determination.

### HIV-1 transmission assay

Raji.DC-SIGN^+^ cells or MDDCs (5x10^5^ cells/200 μl) were exposed to high amounts of HIV-1 HE (100 μl; ∼2.5x10^6^ pg/ml of p24) for 1 h at 37°C. Test compounds were diluted in a 96-well plate (100 μl) and pre-incubated for 30 min. with CD4^+^ C8166 cells (1x10^5^ cells/50 μl). The virus-exposed Raji.DC-SIGN cells or MDDCs were extensively washed, resuspended and cocultivated with the C8166 cells at the same cell density for 48 h. After light microscopic evaluation of syncytium formation, supernatant was collected for p24 HIV-1 Ag ELISA. In addition, the cocultures with Raji.DC-SIGN^+^/HE and C8166 cells were washed and stained with anti-CD4-FITC/anti-CD209-PE (both from BD) as described above. Aspecific binding was determined using SimulTest control (IgGγ1-FITC/IgGγ2a-PE; BD).

### HSV-2 transmission and subsequent replication assay

MDDCs (1x10^6^ cells) were pre-incubated with high amounts of HSV-2 G **(170 PFU)** for 3 h at 5% CO_2_ and 37°C. Afterwards, the cells were extensively washed with PBS/FCS2% and resuspended at a density of 1x10^5^ cells/50 μl in RPMI culture medium. LA (100 μl) was diluted in RPMI culture medium and pre-incubated for 30 min. with CD4^+^ MT-4 cells (1x10^5^ cells/50 μl) in a 96-well plate before being added to the same amount of HSV-2 exposed MDDCs. Five days after cocultivation, giant cell formation was scored microscopically and dying MT-4-infected cells were detected by flow cytometric side-scatter analyses.

### *In vivo* anti-HSV-2 activity of LA in infected mice

The Ethical Committee for Animal Care and Use of the University of Leuven approved all animal work (P221/2014). Animals’ guidelines and policies were in accordance with the Belgian Royal Decree of November 14^th^, 1993 concerning the protection of laboratory animals and the EU Directive 86-609-EEC for the protection of vertebrate animals used for scientific purposes.

Female adult nu-nu mice were treated topically with 50 μl of 2.5% LA, 5% acyclovir, 1% tenofovir (all dissolved in DMSO) or placebo (just DMSO) twice daily for 5 days, starting one day prior to infection. On the day of infection, the skin of the mice was scarificated lumbosacrally and subsequently exposed to a HSV-2 G suspension (500 PFU/50 μl). Development of lesions and mortality were recorded over 25 days. Primary lesions develop at the site of inoculation and the severity of the infection was given a score based on the number and size of the lesions [9, 32]. Initially the lesions are very small (“small early lesions”) and increase in size and number over time (“advanced lesions”). Swab samples were taken from the lesion sites at day 6 and 7 post-infection by firmly rubbing the lesions. The swabs were subsequently immersed in 3 ml PBS and the viral titers of the samples were determined in human embryonic lung fibroblasts [9]. GraphPad Prism 5 was used for developing the figures and performing Mann Whitney U test (GraphPad Software Inc, La Jolla, CA, USA).

### *Lactobacillus* growth assay

The *Lactobacillus* strains used in this study are listed in S1 Table. All strains were routinely grown non-shaking in MRS (de Man-Rogosa-Sharpe) medium (BD Difco) at 37°C [33]. The capacity of different *Lactobacillus* strains to grow in the presence of various concentrations of LA was performed as described previously [34]. Briefly, *Lactobacilli* grown overnight to stationary phase (OD_600nm_ ∼1.8–2.0) were used as inocula for the assay. The assay was performed in 96-well plates. Sterile wells were filled with 200 μl MRS medium. About 0.5x10^7^ colony forming units (CFUs) (1/100 of an overnight culture) were added and various concentrations of LA were administered. The plates were incubated without shaking for 24 h at 37°C before the OD_600nm_ was measured. As a negative control, each strain was grown in compound free medium. Each of the *Lactobacillus* strains and components were examined in 4 replicates and each experiment was independently performed at least 3 times.

### Cellular activation marker expression

The expression of the cellular activation markers CD69 (early), CD25 (late) and HLA-DR (very late) were measured on freshly isolated PBMCs after 3 days of incubation at 37°C with varying concentrations of LA, PRO2000, tenofovir and PHA. Briefly, the cells were washed with PBS/FCS2% and incubated with PerCP-conjugated anti-CD4 mAb (BD Biosciences) together with PE-conjugated anti-CD25 mAb (BD Biosciences), anti-CD69 mAb (Biolegend) or anti-HLA-DR mAb (BD Biosciences) for 30 min. at 4°C. In parallel, the cells were stained with SimulTest control IgGγ1-FITC/IgGγ2a-PE (BD) for aspecific background staining. After washing, the cells were fixed with 1% paraformaldehyde solution and analyzed with the FACSCalibur and Cell Quest software (BD).

### Susceptibility of PBMCs for HIV-1 infection

Freshly isolated PBMCs were cultured in the presence of LA, PHA, tenofovir, PRO2000 and acyclovir for 24 h. The next day, the cells were collected and extensively washed in cell culture medium, before being seeded at a density of 5x10^5^ cells/450 μl in a 48-well plate in the presence of 2 ng/ml IL-2. Afterwards, 50 μl of HIV-1 R5 BaL (TCID_50_ depending on the viral stock) was given and supernatant was collected 7 days post-infection to measure HIV-1 replication by p24 HIV-1 Ag ELISA.

### LA/antiviral drug combination assays

The methodology of analysis of two-drug combinations was described earlier [29, 35]. Shortly, IC_50_s of each inhibitor solely were determined in C8166 cells for HSV-2 G and TZM-bl cells for HIV-1 R5 BaL. Afterwards, viral replication was determined using MTS/PES method in the presence of LA in combination with acyclovir, PRO2000 and LabyA1 for HSV-2. The anti-HIV activitiy of LA in combination with maraviroc, tenofovir and PRO2000 was determined using the luminiscence assay. Combination indices were calculated using CalcuSyn software (Bio-Soft, Cambridge, UK) based on the median-effect principle of Chou and Talalay [36].

### Selection of HIV-1 NL4.3^LAresistant^ virus and genotyping of the HIV-1 *env* region

HIV-1 NL4.3 was added to MT-4 cells in 24-well plates in the presence of increasing concentrations of LA. Every 5 days, viral replication was scored microscopically and compound concentration was increased when full CPE was observed. After 55 passages, HIV-1 NL4.3^LAresistant^ virus was collected and viral RNA was extracted from cell culture supernatants using QIAamp Viral RNA minikit (Qiagen, Hilden, Germany). The genotyping of the *env* gene was determined as described earlier [37].

## Results

### LA has a broad-spectrum anti-HIV activity *in vitro* with low cytotoxicity

**Fig 1:**
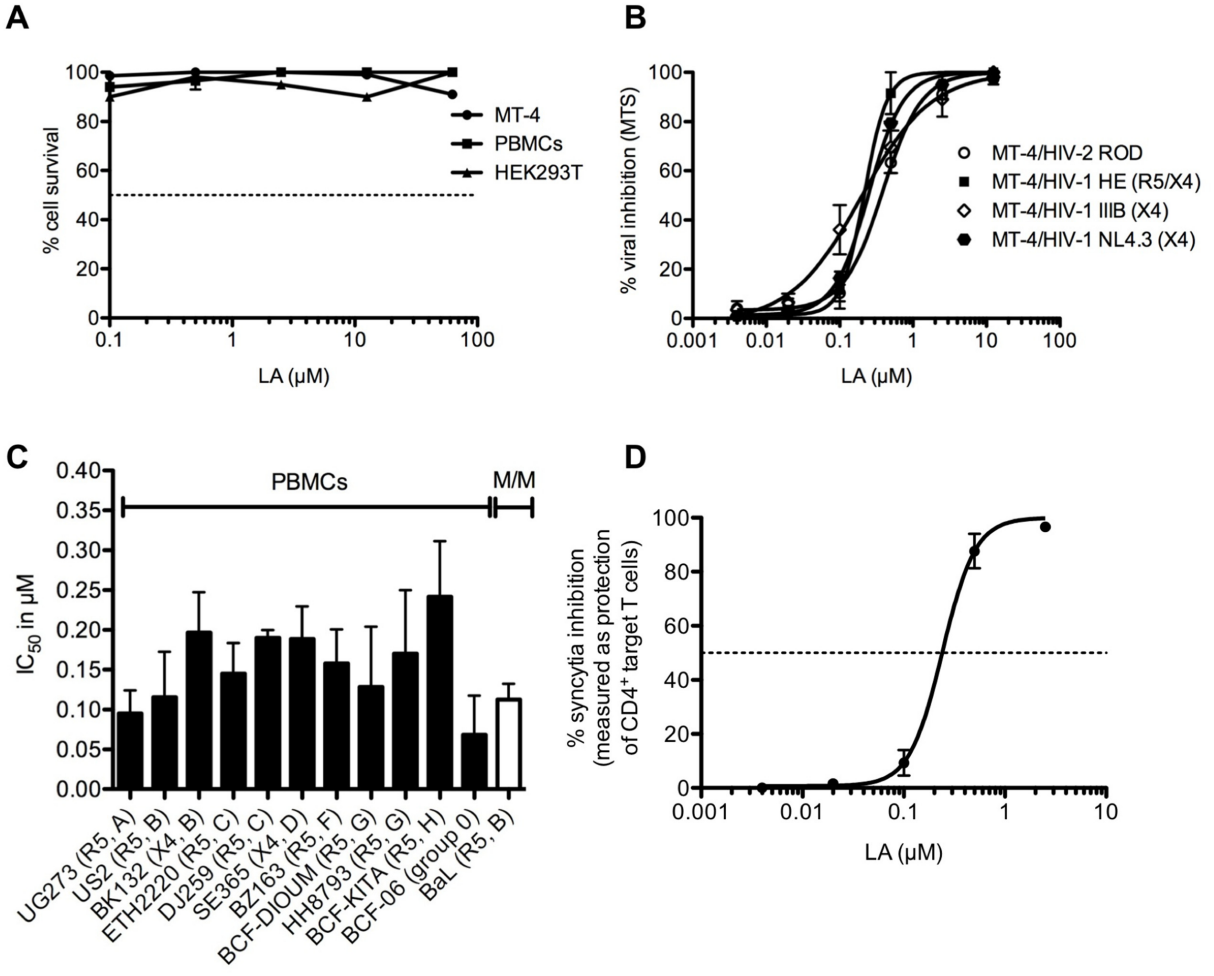
LA has a potent broad-spectrum anti-HIV activity with low, if any, cytotoxicity. (A) Concentrations up to 62.5 μM (500 μg/ml) of LA were given to MT-4 cells, HEK293T cells and PBMCs. Cytotoxicity levels and CC_50_s were determined spectrophotometrically using MTS/PES method. (B) Dose-dependent anti-HIV activity of LA in the CD4^+^ T-lymphoma cell line MT-4 against 3 laboratory HIV-1 strains (NL4.3, IIIB and HE) and 1 HIV-2 strain (ROD) with respectively the following IC_50_s: 0.20 ± 0.03 μM (NL4.3), 0.22 ± 0.06 μM (IIIB), 0.22 ± 0.00 μM (HE) and 0.34 ± 0.04 μM (ROD). Mean IC_50_ ± SEM up to 6 independent experiments is shown. (C). Evaluation of the IC_50_s of LA against various clinical isolates representing different subtypes (A-H), irrespective of HIV-1 coreceptor tropism in primary cells (PBMCs and monocyte/macrophages [MDM]). Viral replication was measured using HIV-1 p24 Ag ELISA with exception of p27 HIV Ag ELISA for BCF-06 (group O). The IC_50_s for viral inhibition were as follows: 0.10 ± 0.03 μM (UG273); 0.12 ± 0.06 μM (US2); 0.20 ± 0.05 μM (BK132); 0.15 ± 0.04 μM (ETH2220); 0.19 ± 0.01 μM (DJ259); 0.19 ± 0.04 μM (SE365); 0.16 ± 0.04 μM (BZ163); 0.13 ± 0.08 μM (BCF-DIOUM); 0.17 ± 0.08 μM (HH8793); 0.24 ± 0.07 μM (BCF-KITA); 0.07 ± 0.05 μM (BCF-06) and 0.11 ± 0.02 μM (BaL). Mean IC_50_s ± SEM from 2–6 independent donor experiments is shown. (D) Dose-dependent effect of LA on the giant cell (syncytia) formation between persistently HIV-1 IIIB-infected T cells (HUT-78/IIIB) and non-infected CD4^+^ target SupT1 T cells. Percentage syncytia inhibition was measured by flow cytometry. Mean ± SEM of 3 independent experiments is shown.

First, we investigated the cytotoxicity of LA in various cell types and as shown in Fig 1A, the 50% cytotoxic concentrations (CC_50_s) were all higher than 62.5 μM (or >500 μg/ml).

Next, the anti-HIV activity of LA against 4 laboratory-adapted strains [2 HIV-1 X4 strains (NL4.3, IIIB), 1 R5/X4 strain (HE) and 1 HIV-2 strain (ROD)] was evaluated in MT-4 cells with IC_50_s ranging from 0.20 to 0.34 μM (1.6–2.7 μg/ml) (Fig 1B). In addition, LA exhibited also a very potent inhibitory activity in PBMCs against various HIV-1 clinical isolates, representing different subtypes (A-H) of group M and in monocytes/macrophages against HIV-1 R5 BaL. The IC_50_s varied between 0.07 and 0.24 μM (0.56–1.7 μg/ml) (Fig 1C). Taken together, these results demonstrate that the antiviral activity of LA is consistent and irrespective of HIV coreceptor tropism.

### Inhibitory activity of LA on HIV-1-associated syncytium formation

HIV infection and transmission is not only characterized by free viral particles, but also by virus-infected cells (e.g. T cells) [38]. Therefore, the antiviral activity of LA was determined using the giant cell (syncytium) assay between persistently HIV-infected T cells (HUT-78/IIIB) and non-infected CD4^+^ target T cells (SupT1 cells). The SupT1 T cells express high amounts of CD28, while HUT-78/IIIB cells do not (data not shown) such that this marker can be used to determine the percentage of SupT1 T cell protection, indicative for the percentage inhibition of syncytium formation. We found that LA neutralized this process very efficiently with an IC_50_ of 0.23 ± 0.02 μM (1.8 ± 0.16 μg/ml) (Fig 1D).

### LA is an HIV-1 entry inhibitor by interfering with viral envelope gp120 binding

**Fig 2:**
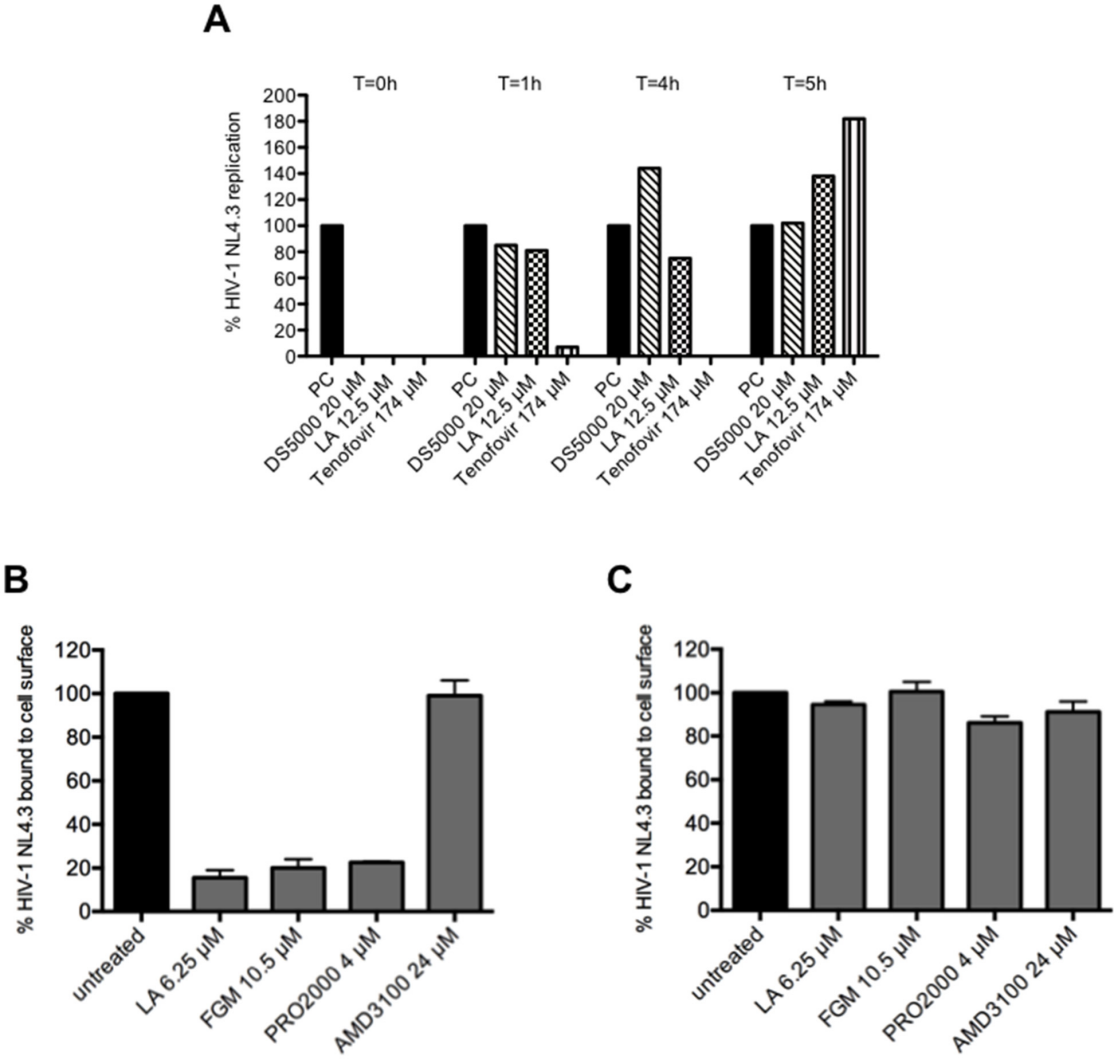
LA binds to the HIV-1 envelope glycoproteins. (A) MT-4 cells were infected with HIV-1 NL4.3 and dextran sulfate, LA and tenofovir were given at different time points post-infection. After 5 h, viral replication was measured using HIV-1 p24 Ag ELISA. One representative experiment out of 3 is shown. (B) The binding of HIV-1 NL4.3 to CD4 on SupT1 T cells in the presence of LA, FGM, PRO2000 and AMD3100, was measured using anti-gp120 9205 mAb. Mean % ± SEM of virus binding to CD4 from 2 independent experiments is shown. *p<0.05 according to student’s T-test. (C) The cells were first pre-incubated for 15’ at room temperature with LA, FGM, PRO2000 or AMD3100, than extensively washed and finally incubated with virus. The binding of HIV-1 NL4.3 to CD4 on SupT1 T cells was again measured using anti-gp120 9205 mAb. Mean % ± SEM of virus binding to CD4 from 2 independent experiments is shown.

To investigate the mechanism of viral inhibition of LA, time-of-drug addition experiments were performed (Fig 2A). The polyanionic compound dextran sulfate (DS5000), a compound known to target HIV-1 gp120 and the NtRTI tenofovir were included as reference compounds. Inhibition of viral infection by LA was completely abrogated 1 h post-infection, while tenofovir kept his antiviral activity up to 4 h (Fig 2A). As can be seen from Fig 2A, LA has a comparable inhibitory profile as dextran sulfate and this finding suggests that LA acts as a HIV entry inhibitor. To provide additional support that LA inhibits HIV-1 entry by interacting with the viral envelope, we performed a virus inactivation assay. As shown in S2 Table, LA had a mild effect on virus inactivation but less compared to the control compounds HHA and aldrithiol.

Based on the time-of-drug addition experiments, we investigated if LA interfered with the initial gp120 binding to the cell surface. Using flow cytometry, virus binding was detected with the anti-gp120 mAb 9205 recognizing the tip of the V3-loop and the 2G12 mAb, which binds to high-mannose type N-glycans that lie around the C4-V4 region on gp120. Similar to FGM (*p* = 0.0318) and PRO2000 (*p* = 0.0041), LA inhibited significantly (*p* = 0.0264) the binding of HIV-1 NL4.3 to the cell surface of SupT1 T cells as detected by the 9205 mAb. Likewise, 2G12 mAb binding was reduced in the presence of LA and this effect was dose-dependent (S2 Fig). As expected the CXCR4 antagonist AMD3100 (*p* = 0.9097) had no activity in the virus binding assay (Fig 2B and S2 Fig).

When we pre-incubated the cells with the above-mentioned entry inhibitors, washed the cells extensively and added the same amount of virus, neither LA (*p* = 0.1695), FGM (*p* = 0.9296), PRO2000 (*p* = 0.1344) nor AMD3100 (*p* = 0.3228) was able to significantly inhibit the binding of HIV-1 NL4.3 to CD4. In summary, these results show that LA binds to the envelope glycoproteins of HIV and presumably not to the cell surface (Fig 2C).

### LA inhibits the DC-SIGN-mediated route of viral transmission

**Fig 3:**
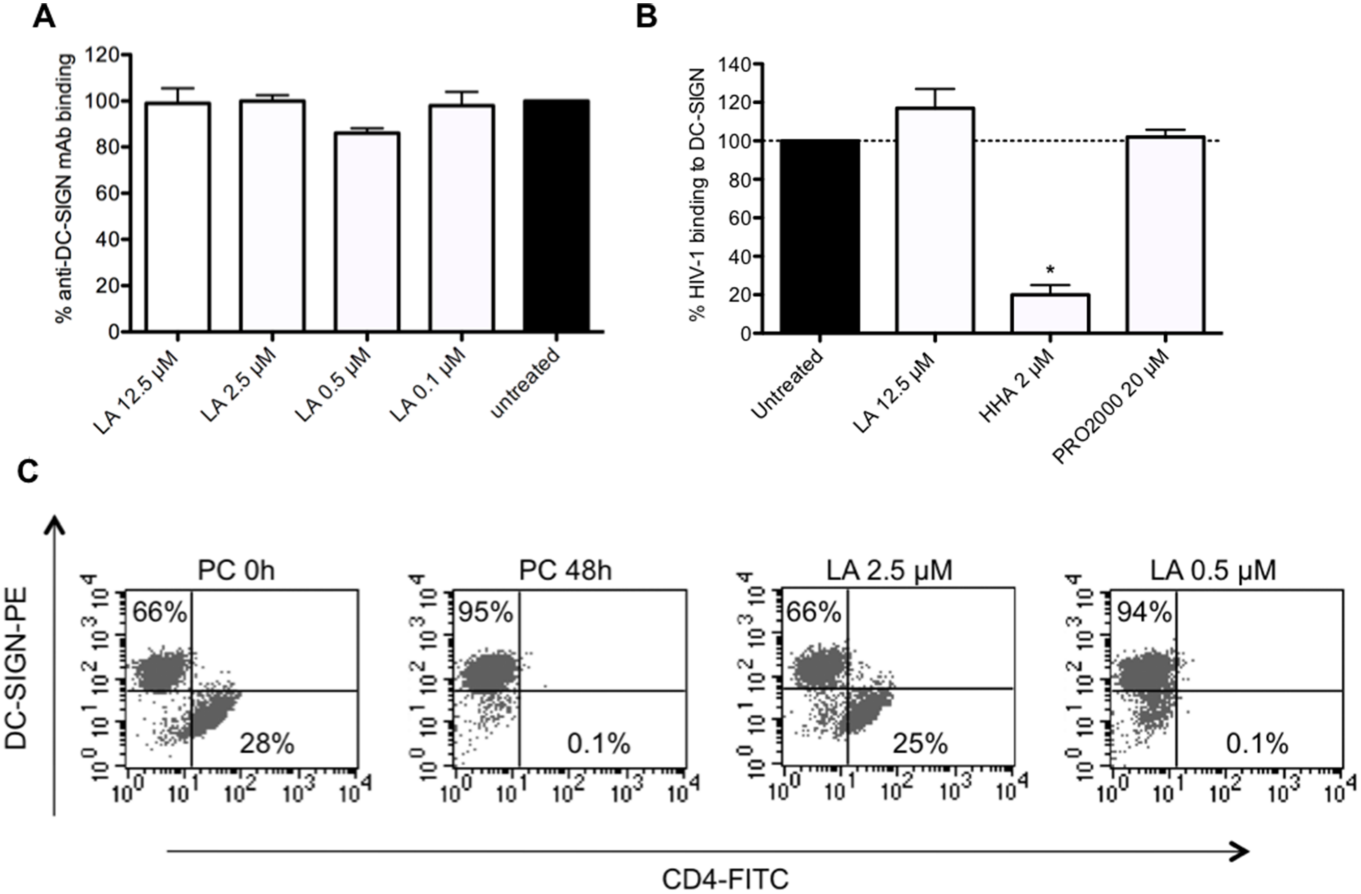
LA inhibits HIV-1 DC-SIGN-related transmission to uninfected CD4^+^ target T cells. (A) Raji.DC-SIGN^+^ cells were pretreated with or without various concentrations of LA for 30 min. The binding of PE-conjugated anti-DC-SIGN mAb (clone DCN46) was measured by flow cytometry. The bars represent the % of anti-DC-SIGN binding relative to the untreated conditions. Mean ± SEM of 3 independent experiments is shown. (B) Effect of LA, HHA and PRO2000 on the capture of HIV-1 strain HE (R5/X4) by DC-SIGN on Raji.DC-SIGN^+^ cells. HIV-1 virions were pre-incubated for 30 min. with the test compounds before being exposed for 1h to Raji.DC-SIGN^+^ cells. After washing the cells, p24 HIV-1 Ag ELISA was used to quantify the amount of cell-associated virus. Bars represent the % virus binding relative to untreated conditions. Mean ± SEM of 3 independent experiments is shown with *p<0.05, according to Student’s T-test. (C) HIV-1 HE (R5/X4) virions were exposed to Raji.DC-SIGN^+^ cells for 1 h. After extensive washing, Raji.DC-SIGN/HE cells were cocultivated with or without compound pretreated uninfected CD4^+^ target C8166 T cells for 48h. Cocultures were stained with PE-conjugated anti-DC-SIGN and FITC-conjugated anti-CD4. The % of positive gated cells in the quadrants of the dot plots is given. One representative experiment out of 3 is shown.

In the next set of experiments, we investigated the binding of LA to DC-SIGN, an important attachment receptor involved in sexual transmission of HIV-1 to CD4^+^ T cells [39]. At the highest concentrations tested (12.5 μM), it appeared that LA was not able to bind to the DC-SIGN receptor, since no dose-dependent inhibition of anti-DC-SIGN mAb was observed (Fig 3A).

Secondly, we pre-incubated high amounts of the R5/X4 HIV-1 strain HE with LA, PRO2000 or HHA prior to exposure to Raji.DC-SIGN^+^ cells. As shown in Fig 3B, the polyanionic compounds LA and PRO2000 did not inhibit the capture of HIV-1 to DC-SIGN, while the carbohydrate-binding agent (CBA) HHA significantly (*p* = 0.0397) interfered with this process.

As DC-SIGN^+^ cells are proposed to be the first host cells facilitating further infection of CD4^+^ T cells in *trans*, we investigated if LA could inhibit the transmission of the R5/X4 HIV-1 strain HE bound to DC-SIGN to the uninfected CD4^+^ target T cells. At the start of cocultivation, ~66% of the viable cells are Raji.DC-SIGN/HE (DC-SIGN^+^/CD4^-^) and 28% are C8166 (DC-SIGN^-^/CD4^+^) T cells (Fig 3C, first panel). After 48 h of coculture, the infected CD4^+^ C8166 target T cells lost CD4 expression and died due to HIV-1 infection (second panel). LA inhibited this process dose-dependently (third and fourth panel) with a mean IC_50_ of 1.09 ± 0.025 μM (8.7 ± 0.2 μg/ml), comparable to the polyanionic compound PRO2000 (IC_50_: 1.2 μM; data not shown). The mean IC_50_-value obtained with the HIV-1 p-24 Ag ELISA was 0.8 ± 0.03 μM (6.2 ± 0.26 μg/ml), which was in the same range as with MDDCs 0.2 ± 0.02 μM (1.6 ± 0.16 μg/ml) (data not shown). The class of CBAs (*e*.*g*. HHA, griffithsin) were previously described to be potent inhibitors of this process [20, 40, 41], as no viral transmission was observed in the presence of 0.79 μM griffithsin (data not shown).

### Potent broad-spectrum anti-HSV activity of LA

**Fig 4:**
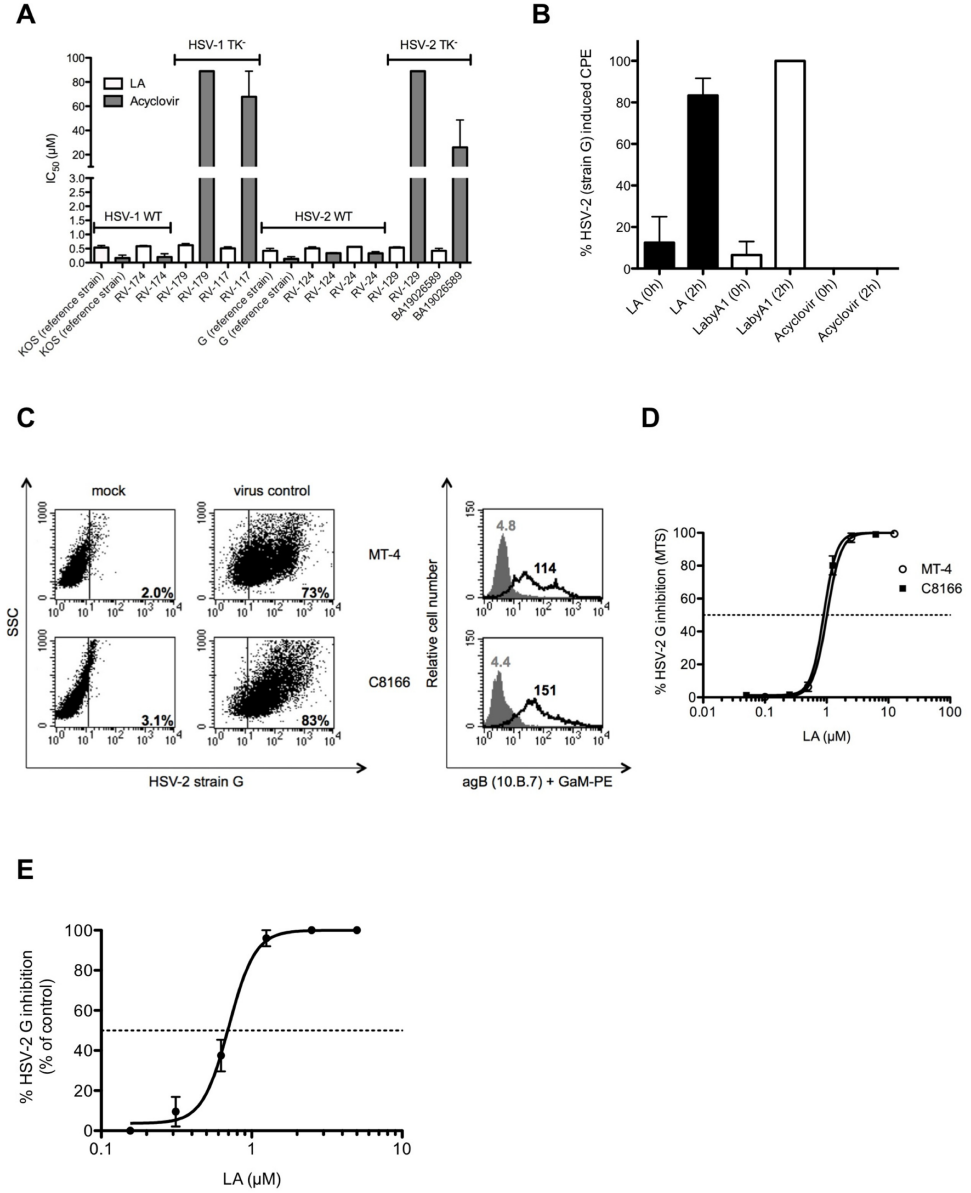
Broad-spectrum anti-HSV-2 activity of LA in epithelial HEL cells, MDDCs and CD4^+^ HIV-susceptible T cells. (A) Dose-depedent anti-HSV activity of LA in HEL fibroblasts against wild-type HSV-1 strains [KOS (reference) and RV-174 (clinical isolate)], TK^-^ HSV-1 strains RV-179 and RV-117 (clinical isolates)], wild-type HSV-2 strains [G (reference), RV-124 and RV-24 (clinical isolates)] and TK^-^ HSV-2 strains (RV-129 and BA19026589 (clinical isolates)]. The following IC_50_s were obtained: 0.53 ± 0.08 μM (KOS strain); 0.59 ± 0.03 μM (RV-174); 0.62 ± 0.06 μM (RV-179); 0.51 ± 0.05 μM (RV-117); 0.42 ± 0.09 μM (strain G); 0.51 ± 0.05 μM (RV-124); 0.56 ± 0.00 μM (RV-24); 0.54 ± 0.02 μM (RV-129) and 0.42 ± 0.09 μM (BA19026589). Mean ± SEM out of 2 independent experiments is shown. (B) HEL cells were infected with HSV-2 G and the compounds [LA (0.5 μM), LabyA1 (9.6 μM) and Acyclovir (10 μM)] were added at the time of infection (0 h) or 2 h post-infection. After 3 days, virus-induced CPE was scored microscopically. Mean ± SEM up to 3 experiments is shown. (C) Infection of HSV-2 G in CD4^+^ HIV-1 susceptible T cell lines. MT-4-, C8166 T-lymphoma cells were infected with HSV-2 G. Percentage of infected cells is indicated after 3 days (MT-4 cells) or 4 days (C8166 cells) post-infection as detected using the mAb [10.B.7] directed to the HSV-2 envelope glycoprotein B (gB) by flow cytometry. Grey histograms represent the background fluorescence, while the black histograms show virus binding. The mean fluoresence intensity (MFI) values are indicated in each histogram. (D) Dose-dependent anti-HSV-2 G activity of LA in the CD4^+^ T-lymphoma cell lines MT-4 and C8166 as measured spectrophotometrically. Mean IC_50_ ± SEM from 5–6 independent experiments is shown. (E) Infection of HSV-2 G in MDDCs. Viral replication was measured 5 days post-infection, relative to the postive control, based on flow cytometry side-scatter analyses. Mean ± SEM of 4 individual donors is shown.

First, we evaluated the broad-spectrum anti-HSV activity of LA using the CPE reduction assay in HEL fibroblasts against various wild-type and acyclovir resistant (thymidine kinase negative [TK^-^]) HSV-1 and HSV-2 strains. LA had a consistent and broad-spectrum anti-HSV activity with an IC_50_ ranging between 0.42 and 0.62 μM (3.4 and 5.0 μg/ml), comparable with acyclovir (IC_50_: 0.13–0.34 μM) (Fig 4A), and remained equally active against a variety of clinical mutant TK^-^ HSV-1 and HSV-2 strains. These mutations resulted in a more than 200-fold decrease in antiviral activity of acyclovir (Fig 4A). Furthermore, inhibitory activity of LA was also found against Canine herpes virus in Madin-Darby Canin Kidney (MDCK) cells with an IC_50_ of 0.53 ± 0.03 μM (4.2 ± 0.24 μg/ml) (data not shown).

To investigate the mechanism of action of LA on HSV-2 infection, we performed once more time-of-drug addition experiments in HEL cells. As shown in Fig 4B, when the compounds were added simultaneously with the virus (0 h), LA as well as the reference compounds LabyA1 (a HSV-2 entry inhibitor) and acyclovir (a DNA polymerase inhibitor) blocked virus infection. When given 2 h post-infection, solely acyclovir was able to inhibit viral replication, while LA and LabyA1 lost most of their antiviral activity (Fig 4B), the latter in agreement with a previous report [31]. These data clearly indicate that LA acts as a HSV entry inhibitor.

Previous research has indicated that besides keratinocytes HSV-2 can also infect T cells and DCs [42–44]. Therefore, we infected two HIV-1 susceptible T-lymphoma cell lines (MT-4 and C8166 cells) with HSV-2 G. Viral replication was revealed with flow cytometry using the mAb anti-glycoprotein B (agB) [10.B.7]. MT-4 (73%) and C8166 (83%) were highly susceptible for HSV-2 infection (Fig 4C). HSV-2 G replication was dose-dependently inhibited by LA in MT-4 and C8166 T cells with an IC_50_ of 1.09 ± 0.05 μM (8.7 ± 0.40 μg/ml) and 0.72 ± 0.07 μM (5.8 ± 0.56 μg/ml), respectively (Fig 4D).

In addition, we found that LA dose-dependently protected MDDCs against HSV-2 G infection with an IC_50_ of 0.71 ± 0.05 μM (5.7 ± 0.40 μg/ml) (Fig 4E).

### LA inhibits the HSV-2 DC-SIGN-mediated route of transmission

**Fig 5:**
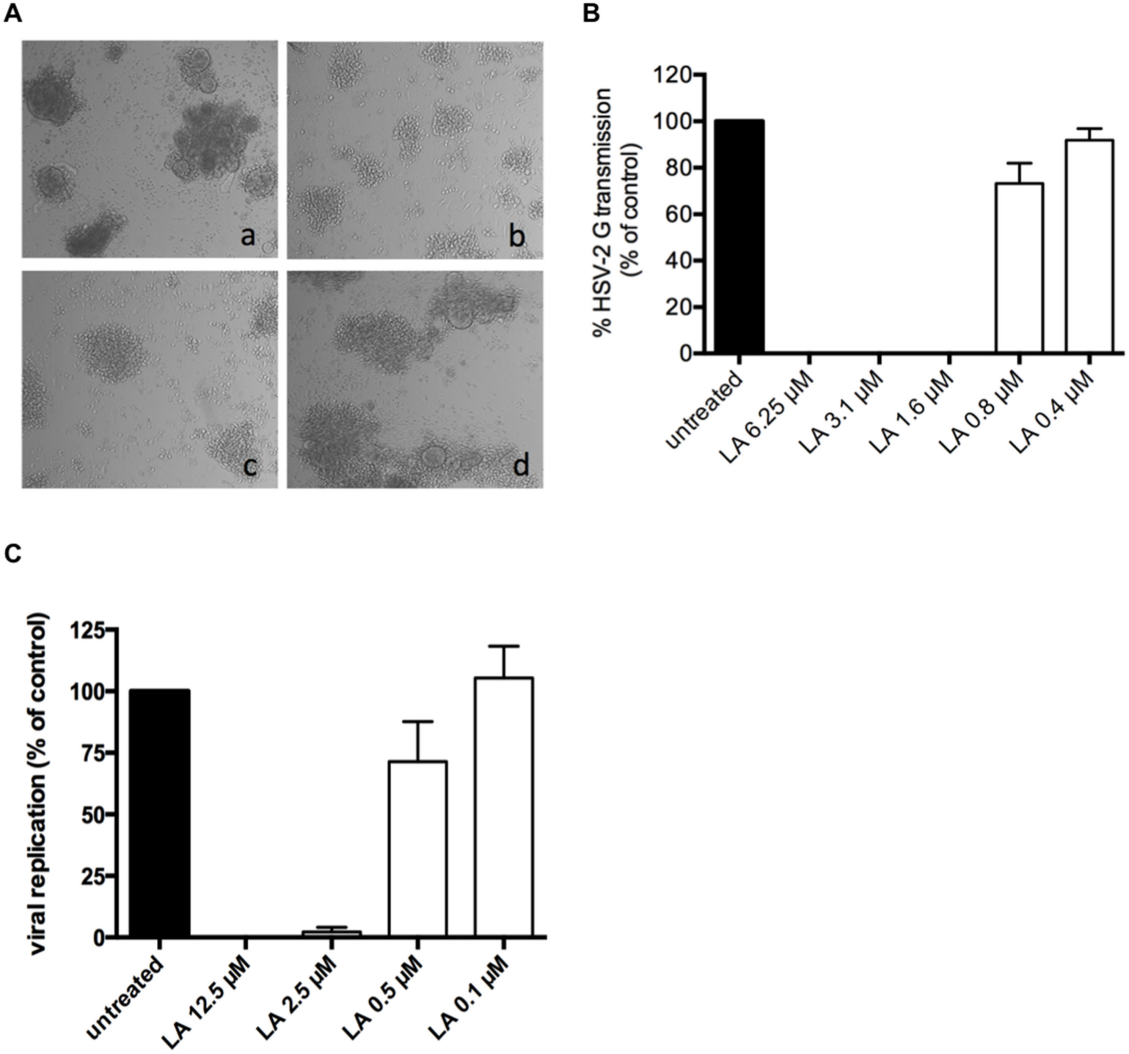
LA inhibits HSV-2 DC-SIGN-related transmission to uninfected target T cells. (A) DC-SIGN^+^ cells were generated out of buffy coat and exposed to HSV-2 G for 2 h. After several washing steps, virus exposed cells were cocultivated with uninfected CD4^+^ MT-4 cells for 5 days. Viral-induced cytopathicity was first scored microscopically. Shown here are the light microscopic pictures of cocultivated HSV-2 G-exposed DC-SIGN^+^ cells and uninfected MT-4 cells 5 days post-cocultivation (a); the effects on HSV-2 G transmission and replication in the presence of 12.5 μM (b), 2.5 μM (c) and 0.5 μM LA (d). One representative experiment out of 4 individual donors is shown. (B) Bars represent the % HSV-2 G transmission and replication in MT-4 cells after 5 days of cocultivation, relative to the positive control. Dying MT-4 infected cells were detected by flow cytometry side-scatter analyses. Mean ± SEM out of 4 individual donors is shown. (C) MDDCs were generated from buffy coat and exposed to HSV-2 G for 2h. After several washing steps, virus exposed MDDCs were co-cultivated with uninfected CD4^+^ MT-4 cells for 5 days. Viral-induced cytopathicity was scored light microscopically and supernatant was retained for further analysis of viral content. Bars represent the percentage of viral replication after 5 days of co-cultivation, relative to the positive untreated control. Mean ± SEM of 3 independent experiments is shown.

Previous studies revealed the importance of the DC-SIGN receptor also in HSV-2 transmission and infection [45]. In the next set of transmission assay experiments, DC-SIGN^+^ cells (MDDCs), derived from buffy coats, were exposed to high amounts of HSV-2 G and after extensive washing cocultivated with CD4^+^ target T cells. After 5 days of cocultivation, massive giant cells were formed (Fig 5A, panel a). At the highest concentrations (12.5 and 2.5 μM (100 and 20 μg/ml) tested, LA inhibited microscopically HSV-2 G transmission and replication completely, while its protective effect disappeared at 0.5 μM (4 μg/ml) (Fig 5A, panels b-d), resulting in an IC_50_ of 1.1 ± 0.1 μM (8.8 ± 0.80 μg/ml). These results are in accordance with our flow cytometry data based on forward scatter (FSC)/side scatter (SSC) changes (IC_50_ of 0.95 ± 0.07 μM) (7.6 ± 0.56 μg/ml) (Fig 5B) and the measured viral titers (IC_50_: 0.8 ± 0.2 μM) (6.5 ± 1.7 μg/ml) (Fig 5C).

### Anti-HSV-2 activity of LA *in vivo*

**Fig 6:**
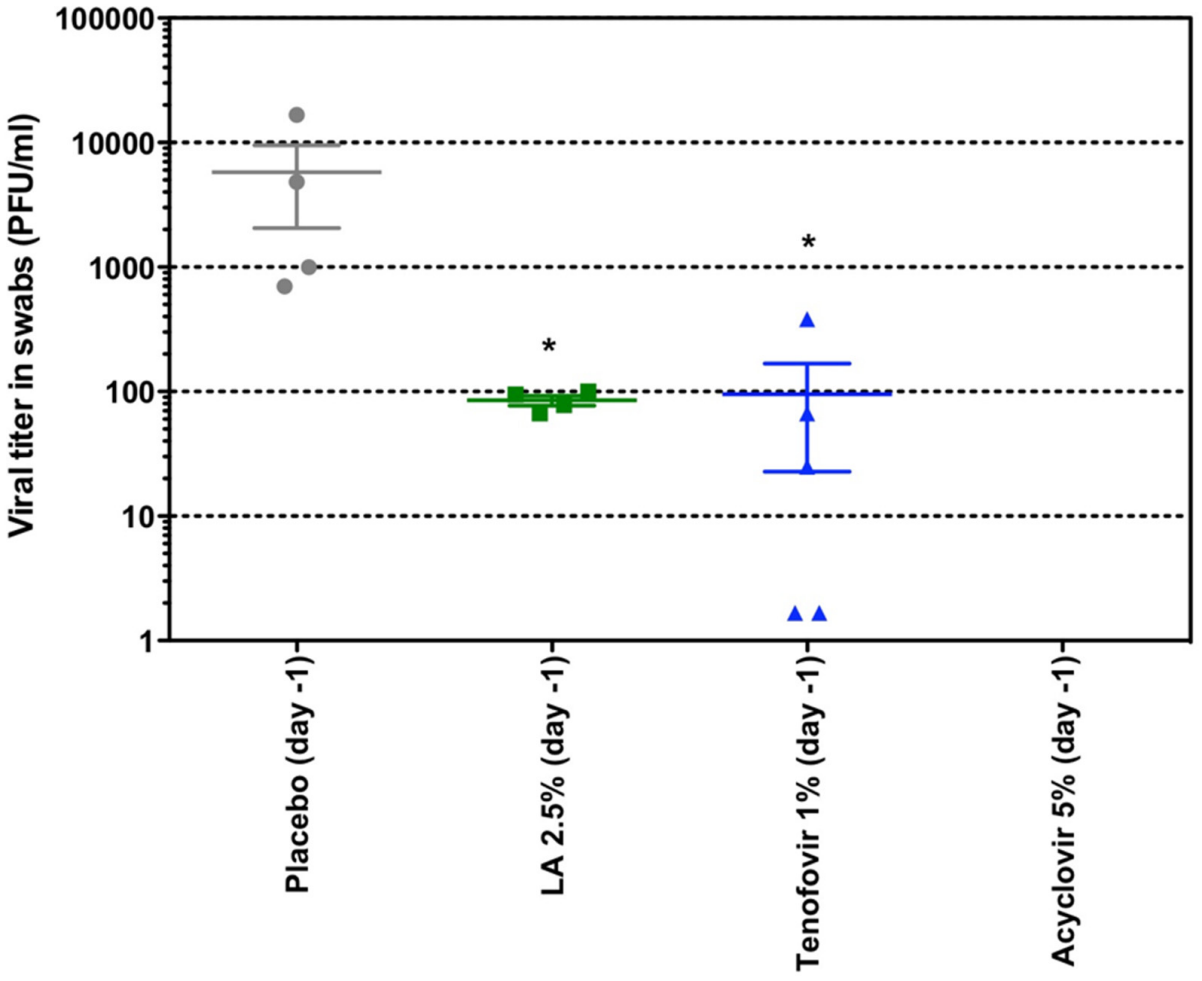
Effects of LA, tenofovir and acyclovir on the viral titers in mice inoculated with HSV-2 G. Groups of five female nu-nu mice were inoculated with HSV-2 on the lumbosacral area. Each group was treated topically two times a day for 5 consecutive days, starting 1 day prior to infection. The placebo cohort received a similar treatment with the test formulation without compound. On day 7 post-infection, swab samples were taken from animals that presented clear advanced lesions or small early lesions. The acyclovir group did not develop any lesions. Mean PFU/ml-values are indicated for each group. Statistical significance compared to placebo: *p<0.05, according to Mann-Whitney U test.

In the next set of experiments, we evaluated the anti-HSV-2 activity of LA *in vivo*. Female nu-nu mice were lumbosacrally scarificated and subsequently inoculated with HSV-2. Each group was treated topically twice daily for 5 days, starting 1 day prior to infection. At days 6 and 7 post-infection, swab samples were taken from the site of inoculation of animals that presented advanced lesions or small early lesion and viral titers of the samples were determined. The highest viral titers were observed in the placebo-arm. Treatment with a solution containing 2.5% LA (*p* = 0.0159) or 1% tenofovir (*p* = 0.0195), both dissolved in DMSO, showed significant lower viral titers compared to the placebo after 7 days (Fig 6). Comparable results were found on day 6 post-infection (data not shown). No viral titers were determined in the acyclovir-treated group, as no lesions were observed. Based on these results we can conclude, firstly, that acyclovir completely suppresses HSV-2 replication and secondly that LA and tenofovir have comparable antiviral effects *in vivo* (Fig 6).

### LA inhibits dual HIV-1 and HSV-2 infection in T cells

**Fig 7:**
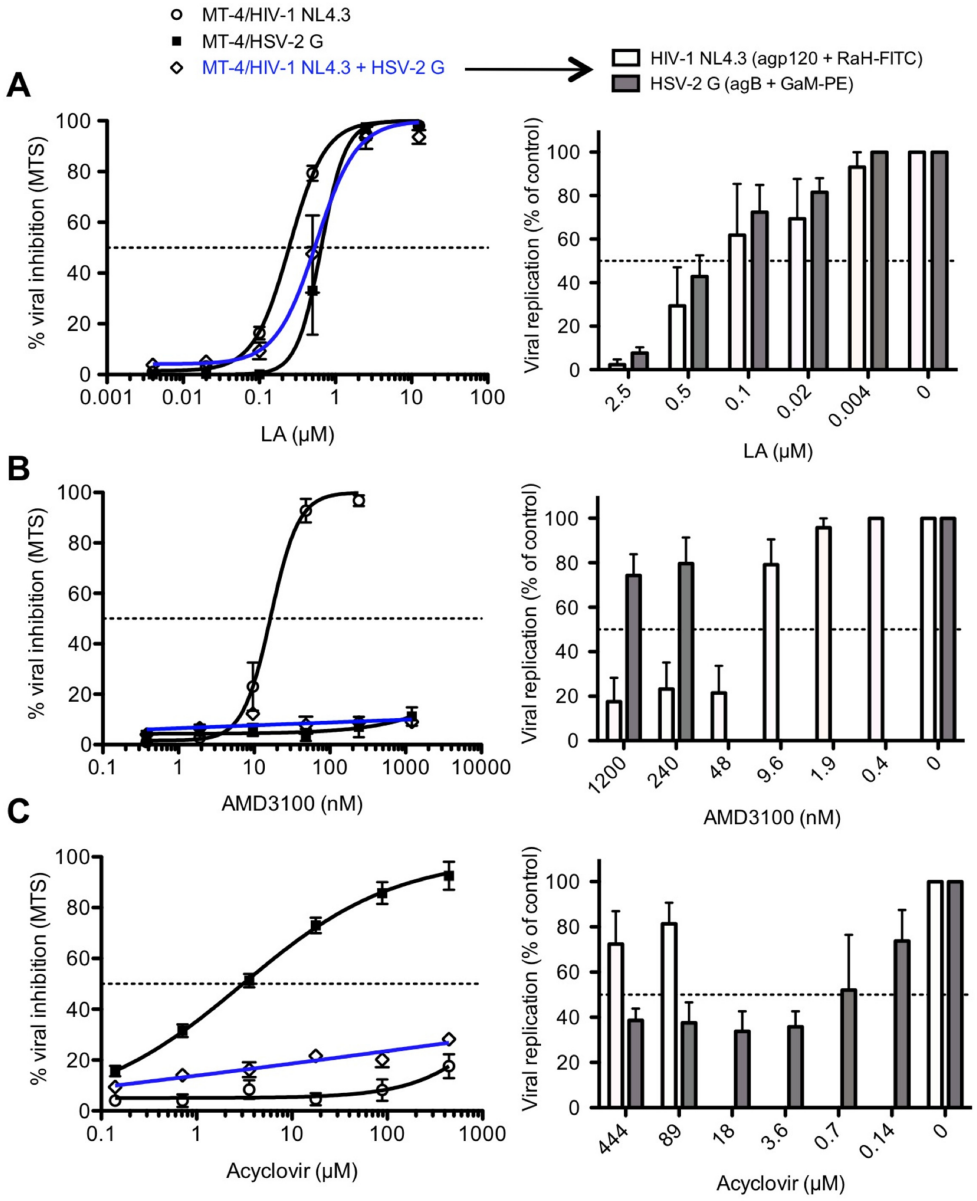
LA inhibits efficiently single and dual HIV-1/HSV-2 infection in T cells. MT-4 cells were infected with HIV-1 NL4.3, HSV-2 G and HIV-1 NL4.3 + HSV-2 G for 3 days in the presence of various concentrations of LA (A), AMD3100 (B) and acyclovir (C). Cytopathicity was measured spectrophotometrically using MTS/PES (left panels). The results are normalized to untreated infected cells. Viral replication at dual infection (shown in blue) was also further investigated by flow cytometry (right panels). HIV-1 NL4.3 replication was detected with b12 mAb + RaH-FITC (white bars) and HSV-2 strain G with agB + GaM-PE (grey bars). Bars indicate the % viral replication in MT-4 cells, compared to untreated conditions. The results shown are the mean ± SEM of 3–5 independent experiments.

The previously described experiments evaluated the effect of LA on the replication of the 2 viruses separately. Next, LA was tested in a cellular HIV-1/HSV-2-co-infection model and its antiviral activity was compared with the well-known HIV-1 entry inhibitor AMD3100 (a CXCR4 antagonist) and the HSV-2 DNA polymerase inhibitor acyclovir. Using the MTS/PES method, we observed that LA dose-dependently inhibited HIV-1/HSV-2 co-infection (IC_50_ = 0.71 ± 0.18 μM (5.7 ± 1.4 μg/ml)) to the same extent as in the single HIV-1 NL4.3 (IC_50_ = 0.20 ± 0.03 μM (1.6 ± 0.24 μg/ml)) or HSV-2 G (IC_50_ = 0.84 ± 0.16 μM (6.7 ± 1.3 μg/ml)) infection conditions, as shown in Fig 7A. Dual HIV-1 NL4.3/HSV-2 G infection was further analyzed with flow cytometry using anti-gp120 mAb b12 for HIV-1 NL4.3 and anti-gB (agB; 10.B.7) mAb for HSV-2 G detection. These anti-HIV-1 NL4.3 (IC_50_ = 0.34 ± 0.18 μM (2.7 ± 1.4 μg/ml)) and anti-HSV-2 G (IC_50_ = 0.50 ± 0.10 μM (4.0 ± 0.8 μg/ml)) flow cytometric data clearly confirmed our observations obtained with the MTS/PES method.

The potent HIV-1 inhibitor AMD3100 lacked anti-HSV-2 activity (IC_50_ >1200 nM), while HIV-1 NL4.3 was dose-dependently inhibited with an IC_50_ of 18.0 ± 2.7 nM (Fig 7B). The MTS/PES method revealed no inhibitory activity of AMD3100 in the dual HIV-1/HSV-2 infection model. However when viral replication of HIV-1 and HSV-2 was measured by flow cytometry using the above-mentioned antibodies, the analysis clearly demonstrated that AMD3100 inhibited solely HIV-1 replication (IC_50_ = 23.4 ± 5.4 nM), as the IC_50_ for HSV-2 was >1200 nM. On the other hand, acyclovir potently inhibited HSV-2 G replication (IC_50_ = 0.85 ± 0.15 μM) in T cells, while lacking anti-HIV-1 NL4.3 activity in single infection conditions. In the co-infection model, no anti-HIV/HSV activity could be detected for acyclovir (IC_50_ >444 μM; Fig 7C) using the MTS/PES method. However, flow cytometric data indicated that acyclovir was still able to inhibit HSV-2 replication in the latter model with an IC_50_ of 1.3 ± 0.9 μM, while having no anti-HIV activity (IC_50_ >444 μM).

### Lack of stimulatory effects of LA on HIV-1 target cells

**Fig 8:**
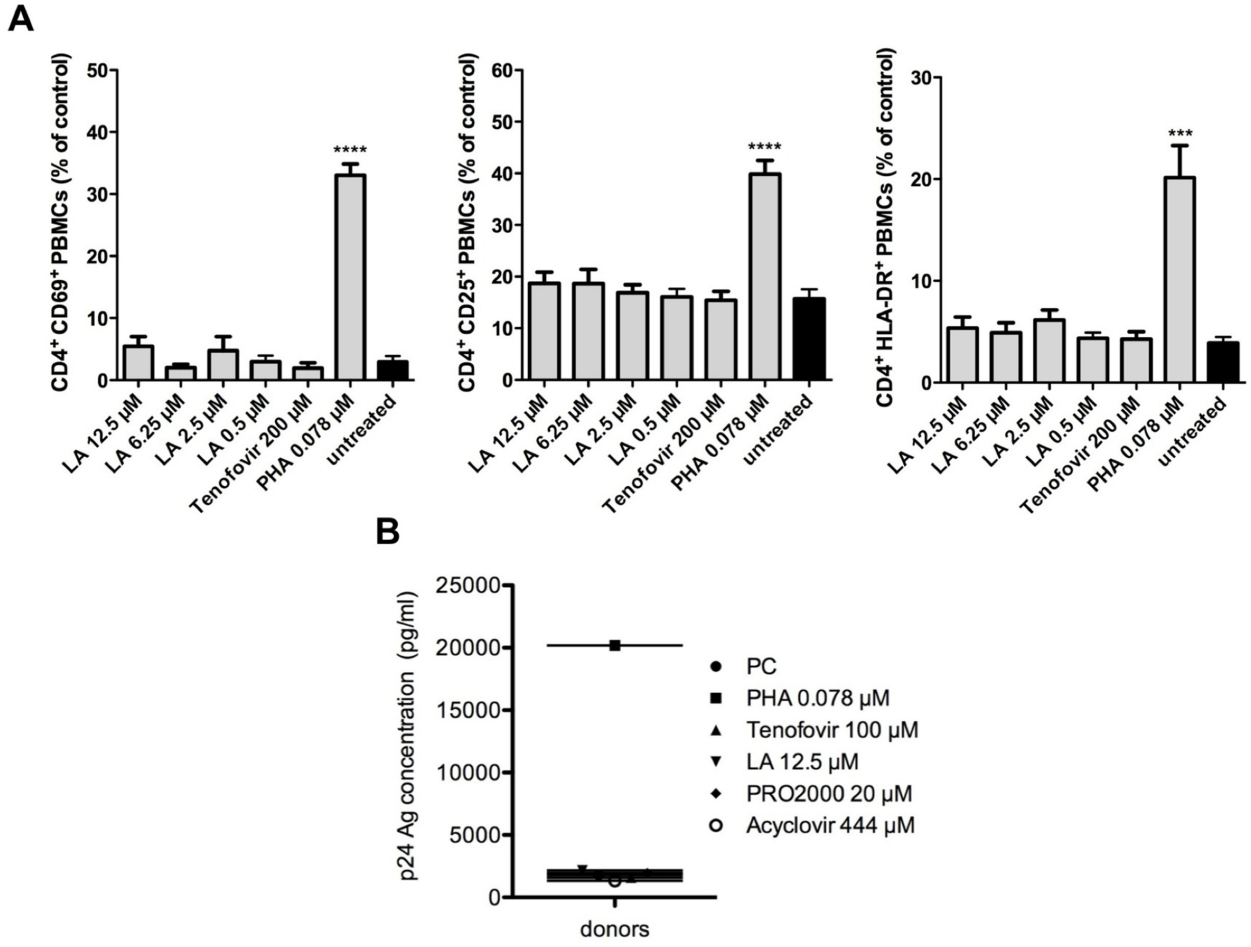
Lack of stimulatory effects of LA on PBMCs. (A) LA does not activate HIV-1 susceptible CD4^+^ T cells. Freshly isolated PBMCs were cultured in the presence of LA, tenofovir or PHA for 3 days at 37°C. The cells were then analyzed for their expression of the activation markers CD69 (early), CD25 (late) and HLA-DR (very late) by the mAbs PE-conjugated anti-CD69 (left), anti-CD25 (middle) and anti-HLA-DR (right) together with PerCP-conjugated anti-CD4. Mean ± SEM of 6 independent experiments is shown. ***p<0.001; ****p<0.0001, according to student’s T-test for comparison with untreated PBMCs. (B) Freshly isolated PBMCs were pretreated with LA, tenofovir, PRO2000, acyclovir or PHA for 24h, then thoroughly washed and infected with HIV-1 R5 BaL for 7 days in the abscence of compounds. Viral replication in the supernatant was measured using HIV-1 p24 Ag ELISA. Mean of 3 independent donor experiments is shown.

To be a potential microbicidal candidate, LA may not activate HIV susceptible target cells. Therefore, LA was thoroughly investigated for its potential cell activating properties. Here, we used the mitogenic lectin PHA as positive control. Freshly isolated PBMCs were exposed to various concentrations of LA, tenofovir or PHA and after 72 h, the expression of the activation markers CD69 (early), CD25 (late) and HLA-DR (very late) on CD4^+^ T cells was determined by flow cytometry. The percentage of CD4^+^CD69^+^ PBMCs in untreated conditions was 2.9 ± 0.9 (mean ± SEM). Treatment with 12.5 μM (100 μg/ml) LA (5.5 ± 1.6%; *p* = 0.1955) or 200 μM tenofovir (1.9 ± 0.9%; *p* = 0.4499) did not significantly increase the number of CD4^+^CD69^+^ T cells. PHA treatment (0.078 μM), on the other hand, markedly increased (11-fold) the amount of CD4^+^CD69^+^ cells (33.0 ± 1.8%; *p*<0.0001) (Fig 8A, left). Compared to untreated conditions (15.7 ± 1.8%) LA had no impact on the number of CD4^+^CD25^+^ expressing T cells (18.7 ± 2.2%; *p* = 0.3251), whereas PHA dramatically increased the amount of CD4^+^CD25^+^ cells (39.8 ± 2.6%; *p*<0.0001) (Fig 8A, middle). In addition, LA did not affect the expression of the very late activation marker HLA-DR. The number of CD4^+^HLA-DR^+^ cells after LA treatment was almost identical to that of the negative control (5.4 ± 1.1% and 3.9 ± 0.6%, *p* = 0.2557; respectively). A significant 5-fold increase (*p* = 0.0005) in the amount of CD4^+^HLA-DR^+^ T cells was observed with PHA (20.2 ± 3.2%; Fig 8A, right).

In the next set of experiments, we determined whether LA treatment of PBMCs for 24 h influenced their susceptibility to HIV-1 infection. Freshly isolated PBMCs from 3 different donors were pretreated with LA, tenofovir, PRO2000 or acyclovir. Once more the mitogenic lectin PHA was included as positive control. After 24 h, the cells were extensively washed and infected with the HIV-1 R5 strain BaL in the absence of compounds. Seven days post-infection, HIV-1 p24 Ag production was determined in the supernatant. The p24 Ag production of untreated cells (PC) was 1806 pg/ml (Fig 8B). Pretreatment with LA did not enhance the cellular susceptibility to HIV-1 considering its p24 Ag concentration of 2152 pg/ml, which was comparable with another polyanionic compound PRO2000 (1947 pg/ml) and the well-known antiviral agents tenofovir (1579 pg/ml) and acyclovir (1321 pg/ml) (Fig 8B). In contrast, pretreatment with PHA led to a dramatic increase in viral replication as demonstrated in p24 Ag levels with values up to 20190 pg/ml.

### No growth inhibitory effects of LA on the vaginal microbiota

**Fig 9:**
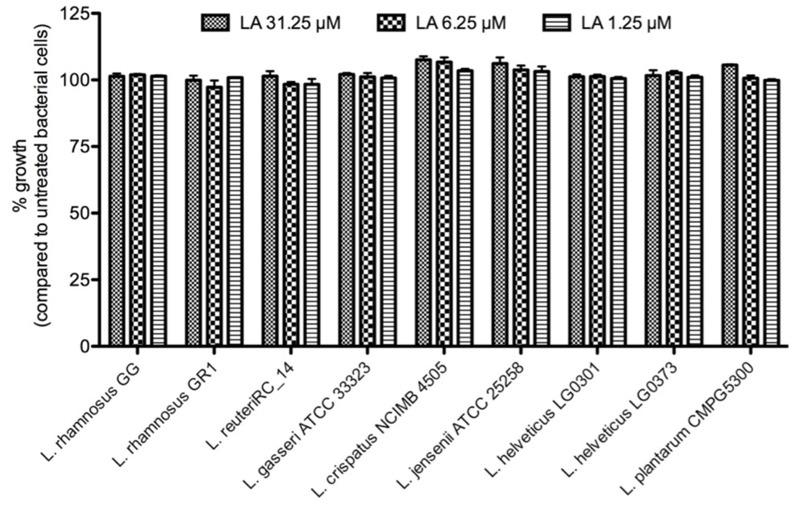
No toxic effects of LA on vaginal *Lactobacilli*. Growth of the gastro-intestinal *L*. *rhamnosus GG* and different vaginal *Lactobacilli* strains in the presence of various concentrations of LA for 24 h. The results are expressed in % of untreated bacterial cells. The mean ± sem of 3 independent experiments is shown.

The vaginal (and also gut) microbiota is very important for the health of the host and may preferentially not be affected by potential microbicidal agents that would offer protection against sexually transmitted diseases such as HIV and HSV [46]. Therefore, we investigated in detail the effects of LA on the growth capacity of the normal vaginal microbiota, which is composed mainly out of *Lactobacillus spp*. All of the tested *Lactobacillus* strains showed the ability to grow in the presence of concentrations of LA as high as 31 μM (or 250 μg/ml) (Fig 9).

### In general synergistic effects of LA in combination assays

**Fig 10:**
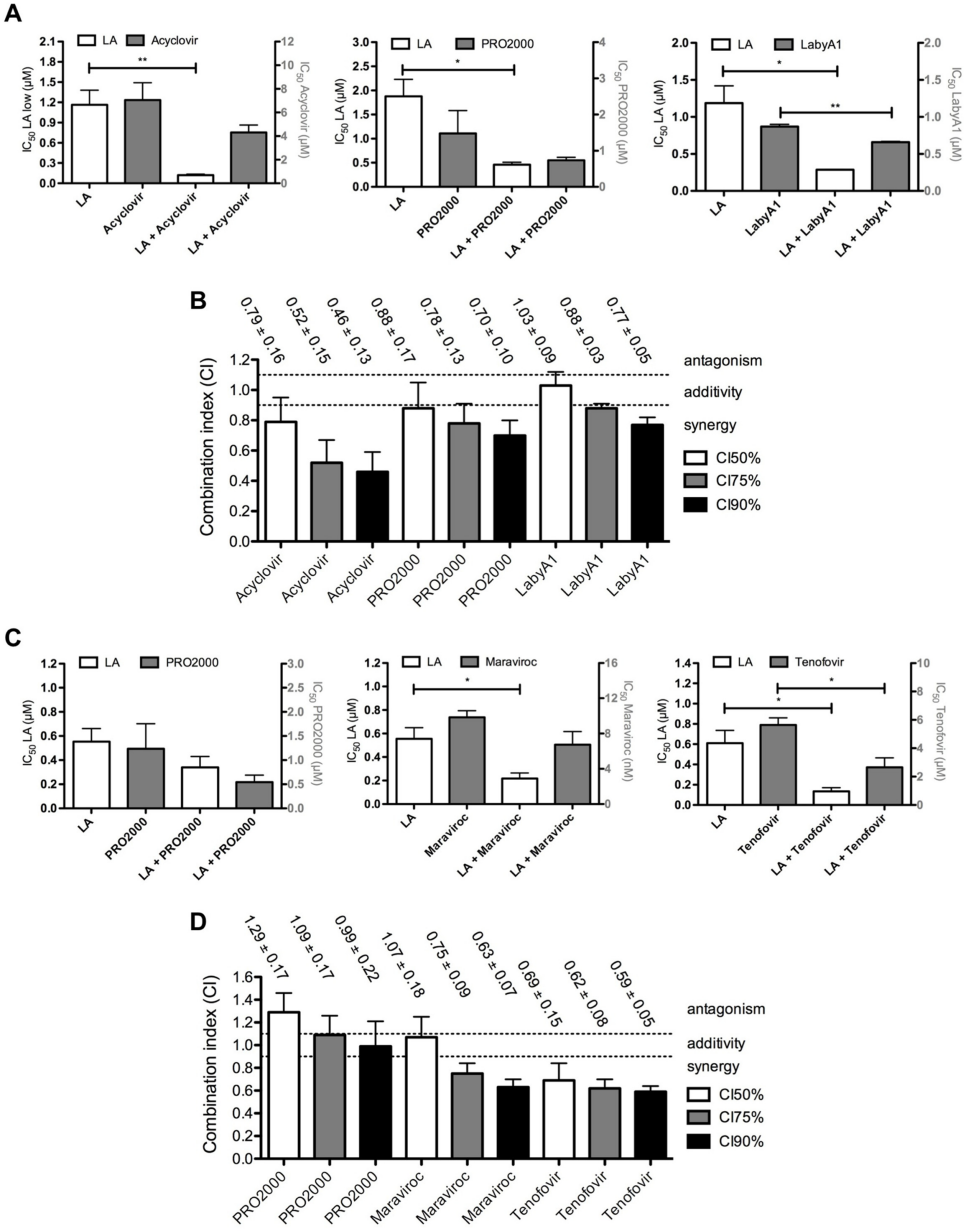
Effects of LA/antiviral drug combinations on HSV and HIV replication. (A) Effects of LA combinations on their anti-HSV-2 G activity in C8166 cells. The IC_50_s of LA (white bars) and the combined inhibitors acyclovir, PRO2000 and LabyA1 (grey bars), alone and in combination, are represented. Mean ± SEM from 3 independent experiments is shown. *p<0.05, **p<0.01, according to student’s T-test, compared with single-drug treatment. (B) Determination of the combination indices (CIs) of LA/acyclovir, LA/PRO2000 and LA/LabyA1 two-drug combinations. Mean CIs ± SEM from 3 independent experiments is shown. The CIs were calculated at 3 inhibitory levels (IC_50_, IC_75_, IC_90_), whereby CIs<0.9 are synergistic, 0.9<CIs<1.1 are additive and CIs>1.1 are antagonistic. (C) Effects of LA combinations on their anti-HIV-1 R5 BaL activity in TZM-bl cells. The IC_50_s of LA (white bars) and the combined inhibitors PRO2000, maraviroc and tenofovir (grey bars), alone and in combination, are represented. Mean ± SEM from 3–4 independent experiments is shown. *p<0.05, according to student’s T-test, compared with single-drug treatment. (D) Calculation of CIs of LA in combination with PRO2000, maraviroc and tenofovir. Mean CIs ± SEM from 3–4 independent experiments is shown. The CIs were again calculated at 3 inhibitory levels (IC_50_, IC_75_, IC_90_), whereby CIs<0.9 are synergistic, 0.9<CIs<1.1 are additive and CIs>1.1 are antagonistic.

An effective microbicide will presumably consist out of at least two different antiviral drugs. Therefore, we investigated if LA acts synergistically with acyclovir, PRO2000 or LabyA1 on HSV-2 G replication in C8166 cells. As shown in Fig 10A, the IC_50_s of LA decreased 10-fold in combination with acyclovir (IC_50_: 0.12 ± 0.02 μM (0.96 ± 0.16 μg/ml); *p* = 0.0089), 4-fold with PRO2000 (IC_50_: 0.46 ± 0.05 μM (3.7 ± 0.4 μg/ml); *p* = 0.0165) or 4-fold with LabyA1 (IC_50_: 0.29 ± 0.01 μM (2.3 ± 0.08 μg/ml); *p* = 0.0183). For each combination with LA, we observed a 1.3- to 2-fold dose reduction, which was only found to be significant when LA was combined with LabyA1 (*p* = 0.0027). CIs were determined to investigate the potential synergistic/additive/antagonistic effects, and as overviewed in Fig 10B, a synergistic interaction was observed with PRO2000 and acyclovir, with the highest levels of synergy in the LA/acyclovir combination. When LA was combined with LabyA1, an additive interaction was observed at the CI_50_-level, but increased synergistic effects were seen with increasing doses (CI_75_ and CI_90_).

In addition, we analyzed the effects of LA in combination with PRO2000, maraviroc or tenofovir on HIV-1 R5 BaL replication in TZM-bl cells. A significant 3-fold decrease in its IC_50_-value was observed for LA in combination with maraviroc (IC_50_: 0.22 ± 0.05 μM (1.8 ± 0.40 μg/ml); *p* = 0.0194) or/and a 4-fold reduction with tenofovir (IC_50_: 0.14 ± 0.04 μM (1.1 ± 0.32 μg/ml); *p* = 0.0221) (Fig 10C). Non-significant dose-reduction effects were observed in combination with PRO2000 (*p*>0.05).

CI analysis revealed antagonistic to additive effects in combination with PRO2000, while synergy was seen at each inhibitory level with tenofovir (Fig 10D). LA/maraviroc combinations showed additivity at CI_50_-levels and increasing synergistic effects with increasing doses.

### Inhibitory effects of LA independent on viral input levels

The amount of infectious virions at the time of transmission is not exactly known. Here, we investigated if LA is able to inhibit HIV-1 infection and replication at increased viral inputs (Table 1). These data clearly indicate that LA kept consistently its anti-HIV activity, with only a 1.8-fold decrease in its IC_50_, even at a 50-fold higher HIV-1 NL4.3 concentration. The other polyanionic compound PRO2000 showed a 4.5-fold decrease. Enfuvirtide (T20), used as reference compound was completely inactive at the highest concentrations tested. In addition, a 3.5-fold increase in IC_50_ for LA was observed with increasing concentrations of HIV-2 ROD, while T20 lacked all anti-HIV-2 activity (data not shown).

**Table 1:** Effects of antiviral activity on increased viral input in MT-4 cells.

	HIV-1 NL4.3 viral input^b^					
	25	125	250	625	1250	
Agent^a^						fold change^c^
LA	0.24 ± 0.02	0.30 ± 0.01	0.28 ± 0.03	0.39 ± 0.04	0.43 ± 0.06	1.8
T20	0.031 ± 0.005	0.099 ± 0.015	0.22 ± 0.05	0.75 ± 0.11	>1.1	>36
PRO2000	0.13 ± 0.03	0.39 ± 0.01	0.46 ± 0.06	0.54 ± 0.05	0.59 ± 0.02	4.5

^a^ Concentrations are in μM.

^b^ Viral input is expressed in pg/well, with 25 pg/well as our reference viral input.

^c^ Fold change in IC_50_s: highest IC_50_/lowest IC_50_.

### Selective mutations in the envelope gene of HIV-1 NL4.3^LAresistant^ virus

**Fig 11:**
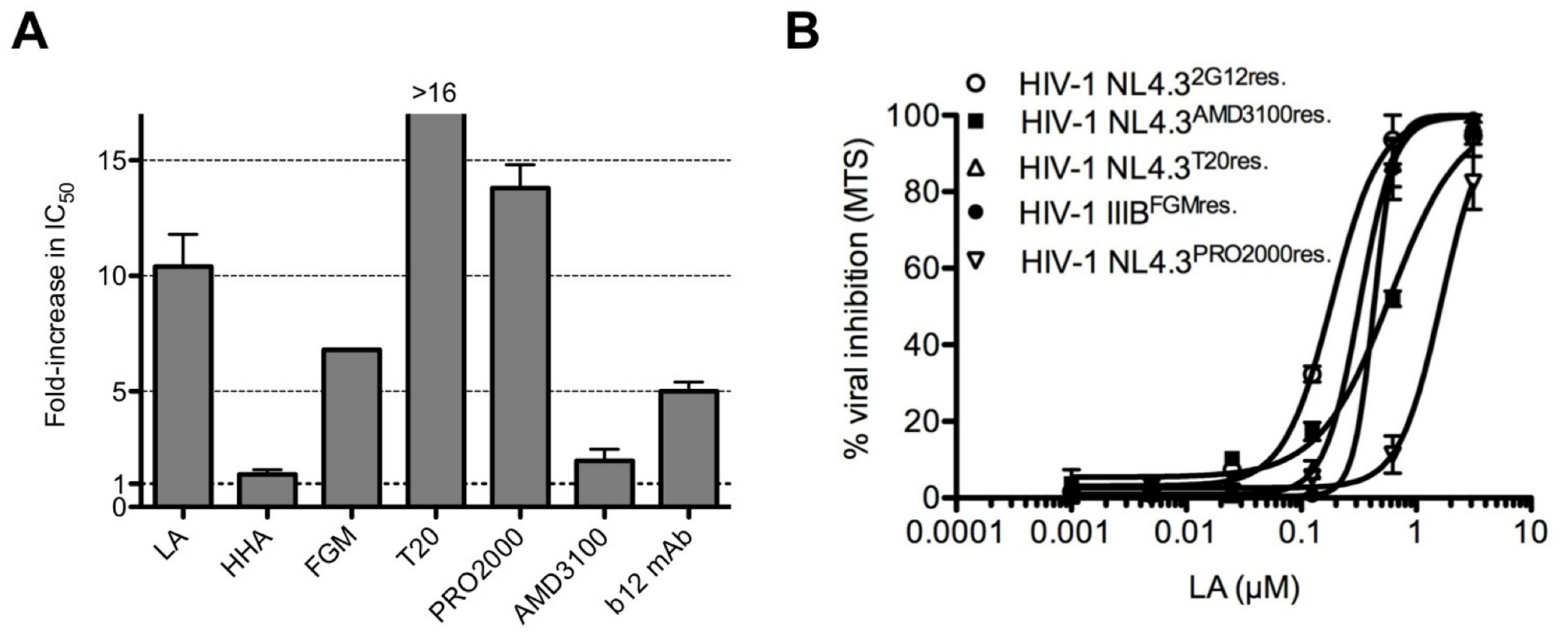
Cross-resistance pattern of the HIV-1 NL4.3^LAresistant^ virus. (A) Fold-increase in IC_50_ of the HIV-1 NL4.3^LAresistant^ virus against the entry inhibitors HHA, FGM, T20, PRO2000, AMD3100 and b12 mAb. Mean ± SEM of 3–4 independent experiments is shown. (B) Dose-dependent effects of LA on the replication of the *in vitro* generated HIV-1 entry inhibitor resistant strains in MT-4 cells. Mean ± SEM of 3 independent experiments is shown.

We generated *in vitro* an NL4.3^LAresistant^ HIV-1 strain to gain more insight in the mechanism of action of LA. Wild-type NL4.3 was cultured in MT-4 cells in the presence of increasing concentrations of LA. As a control, wild-type NL4.3 virus was subcultivated in parallel in the absence of compound. The HIV-1 NL4.3^LAresistant^ virus acquired four mutations throughout gp120: S160N in the V2 loop, V170N in the C2 region, Q280H in the V3 loop and R389T in the C5 region. Two additional mixed mutations were found at R118K/R and A310A/V. In gp41, 3 mutations were observed: K77Q adjacent to HR1 domain, N113D, which belongs to glycosylation sequon “NXT” and H132Y inside HR2 domain. The HIV-1 NL4.3^LAresistant^ virus showed a 10.5-fold decrease in sensitivity to LA (Fig 11A).

### Cross-resistance of HIV-1 NL4.3^LAresistant^ virus against various HIV entry inhibitors

As shown in Fig 11A, high levels of cross-resistance were observed with viral envelope binding agents FGM (7-fold), T20 (>16-fold), PRO2000 (14-fold) and mAb b12 (5-fold), as shown with increased IC_50_-values, while the antiviral activity of HHA and AMD3100 remained unchanged.

### LA showed no cross-resistance with various HIV entry inhibitor resistant strains

As LA acts as an entry inhibitor, potential cross-resistance was further evaluated against 5 HIV-1 entry inhibitor resistant strains (Fig 11B) that were obtained by targeting different steps in the HIV-1 entry process. LA inhibited the wild-type HIV-1 strains NL4.3 and IIIB with an IC_50_ of, respectively, 0.20 and 0.22 μM (1.6 and 1.8 μg/ml) (Fig 1B). No significant loss in antiviral activity was observed against the HIV-1 strains resistant to 2G12 mAb (0.20 ± 0.00 μM (1.8 ± 0.00 μg/ml); NL4.3^2G12res^.), FGM (0.33 ± 0.03 μM (2.6 ± 0.24 μg/ml); HIV-1 IIIB^FGMres^.), AMD3100 (0.57 ± 0.05 μM (4.6 ± 0.40 μg/ml); NL4.3^AMD3100res^.) and T20 (0.30 ± 0.02 μM (2.4 ± 1.6 μg/ml); NL4.3^T20res^.). Only a significant ∼8-fold decrease (*p* = 0.0185) in antiviral activity (IC_50_: 1.5 ± 0.2 μM (12 ± 1.6 μg/ml)) of LA was observed against the NL4.3^PRO2000res^. HIV-1 strain.

## Discussion

In this study, we investigated the antiviral activity and microbicidal properties of the novel low-cost sulfonated polyanionic compound, lignosulfonic acid (LA).

LA demonstrated a potent and consistent broad-spectrum anti-HIV activity in the lower μM-range with low, if any, cytotoxicity in replication and cocultivation assays (Fig 1). These findings are in accordance with recently published data [18], where Qui and colleagues demonstrated the binding of a sulfonated lignin to the V3 loop of gp120 [18]. This loop plays a critical role in the entry process of HIV by interacting with the chemokine receptors CCR5 and/or CXCR4 [47]. This observation could explain its broad-spectrum anti-HIV activity against both R5 and X4 viruses. However, in the past, many concerns have been raised as X4 and R5 HIV-1 strains differ substantially in their sensitivities to polyanionic compounds, due to differences in positive charges [48]. As indicated for PRO2000 [49], LA inhibited equally well X4 and R5 viruses.

HIV-1 binding experiments demonstrated that LA, just like the included reference compounds FGM [24] and PRO2000 [50], significantly inhibited the initial interaction of free HIV-1 virions to the cell surface of target CD4^+^ T cells (Fig 2B). These results correspond to those obtained with other water-soluble lignins using rgp120 and sCD4 [18]. We hypothesize that this reflects inhibition by LA of the interaction between gp120 and cellular CD4. Nevertheless, we also need to consider the possibility that LA compromises virion binding to other T cell surface molecules such as heparan sulfate proteoglycans [51]. As a washing step after pre-exposure of the CD4^+^ T cells to compounds (i.e., LA, PRO2000, FGM) did not prevent virus binding, the interaction between LA and the cell surface appears to be rather weak (Fig 2C). These findings led us to believe that LA mainly binds to the HIV-1 envelope glycoproteins and not to the cell surface. Nevertheless, we (data not shown) and others observed a dose dependent binding inhibition of anti-CD4 mAb binding to the CD4 receptor in the presence of LA variants [18]. This could represent an additional mode of interference with gp120-CD4 interaction. However, this anti-CD4 mAb binding inhibition was only observed in the high LA concentration range (100–200 μg/ml) [18].

Epitope mapping experiments using the neutralizing mAbs 2G12, targeting the N-linked glycans on gp120; b12, recognizing the CD4 binding site on gp120; and 447-52D, binding to a part of the V3 loop on gp120; indicated that a certain sulfonated lignin significantly inhibited dose-dependently the antibody binding to the CD4 binding site and the V3 loop on gp120 [18]. These findings suggest that LA interacts with multiple sites on gp120. In addition, we generated an *in vitro* LA-resistant HIV-1 strain. The observed mutations throughout gp120 gave indeed another indication that LA interacts with different regions on gp120. We found that LA is able to interfere with the CD4/gp120 binding as the V170N mutation occurred in a sequence of the C2 region, which is part of the CD4-induced epitope [52]. The R389T substitution in the C5 region is situated adjacent to the V4 loop and was described previously as another important sequence involved in CD4 and mAb b12 binding [52, 53]. The mutation Q280H on the tip of the V3 loop explains the interaction of LA with the V3 loop and this mutation was also observed with previously reported polyanionic compounds [54]. The mutations around the CD4 binding motifs and in the V3 loop could explain the observed cross-resistance with PRO2000 and FGM (Fig 11), two compounds described to inhibit CD4/gp120 binding [24, 49, 50]. The moderate cross-resistance with b12 mAb, which targets the CD4-binding site on gp120, could be explained by the observed mutation at one of the b12 mAb contact sites and a reported inhibition of b12 mAb by a sulfonated lignin for binding to gp120 by Qui *et al*. [18]. The lack of 2G12 mAb binding inhibition by this lignin on gp120 [18] also corresponds with our findings, as no high-type mannose N-linked glycans were deleted on gp120. On that account, the mannose-specific CBA HHA kept its full antiviral activity (Fig 11) in our cross-resistance studies. Finally, the K77Q (adjacent to HR1) and H132Y (in domain HR2) mutations, both in gp41, reduced the activity of T20, the FDA-approved gp41 fusion inhibitor. Although the cross-resistance of a LA-resistant strain to the only FDA-approved fusion inhibitor might seem cumbersome, the use of T20 in daily practice is drastically reduced due to the fact that it needs to be injected twice daily and because of the continuous emergence of T20 resistant mutations [55–57].

Previous studies highlighted the clinical importance of genital HSV-2 infections as a co-factor for increased HIV transmission in women [2, 6, 58]. We showed for the first time that LA possessed a comparable antiviral activity (lower μM-range) against wild-type and acyclovir-resistant HSV-1 and HSV-2 strains (Fig 4A). These observations are very important regarding the occurrence of acyclovir-resistant strains not only in immunocompromised patients, but also in immunocompetent individuals [11, 59]. Another important observation is that LA could also inhibit HSV-2 infection in MDDCs, one of the key cells of the innate immune system involved for T cell responses. Additionally, MDDCs could also contribute to viral spread in the mucosa [60]. HSV-2 infection results in the expression and secretion of retinoic acid, which induces the expression of α4ß7 integrin on CD4^+^ T cells to make them more vulnerable for HIV-1 infection [44]. Recently, Stefanidou *et al*. demonstrated that HSV-2 infection prevents MDDCs maturation, induce apoptosis and triggers the release of pro-inflammatory cytokines, which could also increase the rate of HIV-1 infection [61]. Here, we also showed for the first time that LA had no effect on the DC-SIGN (or Dendritic cell-specific ICAM-3 grabbing non-integrin) receptor (Fig 3). DC-SIGN acts as a pathogen recognition receptor for viruses and bacteria [62]. Some pathogens, such as HIV and HSV, are captured by DC-SIGN and misuse its function [63] so that they are subsequently transmitted to uninfected target cells [40, 45]. We could demonstrate that both viruses are dose-dependently inhibited by LA in DC-SIGN-related cellular transmission pathways (Figs 3 and 5).

As LA is a highly negatively charged compound, it cannot cross the cellular membrane and based on the HSV-2 time-of-drug addition experiments (Fig 4B) we believe that LA binds to one or more envelope glycoproteins of HSV. These *in vitro* broad-spectrum antiviral activity data indicate that LA acts as a dual HIV/HSV entry inhibitor (Figs 2 and 4) with equal potency against both viruses. Another indication that LA targets the envelope proteins is the lack of antiviral activity of LA against non-enveloped viruses such as the *Picornaviridae* Coxsackie type B4 and the *Reoviridae* Reovirus type 1 (>100 μg/ml or 12.5 μM; data not shown). These findings are in accordance with those described for other polyanions [64].

Acyclovir has been described to possess anti-HIV activity by acting as an reverse transcriptase inhibitor in herpes virus negative cells [13]. Other research groups found such inhibitory activity only when certain herpes viruses were already present and pre-established an infection in CD4^+^ T cells [12]. The lack of activity of acyclovir against HIV in our experimental co-infection model could be explained by the fact that we infected the CD4^+^ T cells with both viruses simultaneously. However, these inconsistent data of acyclovir on HIV inhibition, rendering it not an ideal molecule for microbicidal applications, unless used in combination with other anti-HIV agents. Therefore, we investigated LA in combination with other antiviral inhibitors. Additive to moderate synergistic effects were observed against HSV-2 infections with PRO2000, acyclovir and the recently described potent lantibiotic HIV/HSV peptide inhibitor LabyA1 [31]. Additivity to synergy was observed with maraviroc and tenofovir against HIV-1 replication, while antagonistic to additive effects were observed with PRO2000. This discrepancy between synergy and antagonism with the LA/PRO2000 combination, could be explained by the differences in envelope glycoproteins of HIV and HSV and the cross-resistance profiles of both polyanions to HIV (Fig 11). To date, tenofovir, applied as a 1% vaginal gel, still is the only drug that significantly inhibited HIV-1 transmission by 39% overall and of HSV-2 by 51% and this even at suboptimal concentrations [8]. The potent inhibitory activity against HIV and HSV-2, together with the observed synergy (and additivity) with tenofovir and other anti-HIV and anti-HSV drugs, makes us believe that LA is a strong microbicide candidate. Especially, as Agarwal and colleagues demonstrated also the feasibility of conjugating chemically nucleoside reverse transcriptase inhibitors with cellulose sulfate, displaying antiviral and contraceptive properties [65].

In addition, cellulose sulfate, which failed throughout clinical microbicide trials, and polystyrene sulfonate combine antiviral activity against HIV-1 and HSV-2 with contraceptive activity [66]. Tollner *et al*., [67, 68] demonstrated that LA also exhibits contraceptive properties as *in vitro* fertilization of macaque oocytes was blocked when sperm was treated before or after capacitation. Importantly, LA (Fig 9), as well as polystyrene sulfonate, did not harm the vaginal microbiota, but the anti-HIV activity of polystyrene sulfonate was not so potent, indicating superior microbicidal properties for LA [69]. As a vaginal or rectal microbicide may not be toxic nor stimulate HIV target cells, we showed that LA did not activate PBMCs, nor increased their susceptibility for HIV-1 infection as our control agents tenofovir, PRO2000 or acyclovir (Fig 7). In contrast, application and removal of cellulose sulfate and carrageenan, resulted in an enhanced subsequent HIV-1 infection *in vitro* [70]. Further studies pointed out that cellulose sulfate altered the epithelial architecture by affecting junctional proteins (e.g. zona occludens 1 (ZO-A1)) [71, 72]. Interestingly, Qui *et al*., mentioned that a water-soluble sulfonated lignin had no effects on the epithelial integrity on different epithelial monolayers [18]. Previously described *in vitro* and *in vivo* data demonstrated a good safety profile of PRO2000 [73, 74]. Our *in vivo* experiments in mice indicated that LA did not show superiority over acyclovir in suppressing HSV-2 infection, however LA demonstrated a significant antiviral effect comparable with tenofovir [9]. We could state, for the first time, that LA demonstrated an anti-HSV-2 activity *in vivo*, evaluating its effect on lesion viral titers (Fig 6). However, the more moderate anti-herpetic activity *in vivo*, compared to its potency *in vitro* could be attributed to the administration route or its formulation. Previous studies in mice, using vaginally applied PRO2000 in solution and a gel-formulation, demonstrated protective effects to genital HSV-2 infection [75]. Further optimizations of *in vivo* studies are planned in the near future using a gel-containing LA preparation.

In conclusion, these *in vitro* and *in vivo* results of inhibition of HIV and HSV transmission and infection by LA, together with its high scale availability and safety profile, make it a very promising microbicidal candidate.

## Supporting Information

S1 Fig Specific cell markers for monocyte-derived dendritic cells (MDDCs). The indicated cell surface markers highlight the purity of the MDDC population used in our experiments. The white histograms show the background fluorescence. The filled grey histograms indicate the expression levels of six cell surface markers, specific for MDDCs after treatment with the growth factors IL-4 and GM-CSF.
(DOCX)

S2 Fig HIV-1 binding to the cell surface of CD4^+^ T-cells in the presence of LA. SupT1 cells were incubated with a high amount of HIV-1 NL4.3 virus (positive untreated control) and combined with or without compounds for 2 hours at RT. Thereafter, cells were extensively washed and gp120 binding was evaluated in all the virus treated conditions with the anti-human 2G12 mAb + RaH-IgG-FITC. The bars represent the percentages of anti-gp120 binding relative to the positive control (d). Each value represents the mean ± SEM of 3 independent experiments. * p<0.05, ** p<0.01, *** p<0.005, *** p<0.001 compared to the nontreated control, according to the one-way Anova and Dunnett’s multi comparison post-hoc test.
(DOCX)

S1 Table *Lactobacillus* strains used in this study. (DOCX)

S2 Table Virus inactivation of the laboratory-adapted NL4.3 strain in MT-4 cells. (DOCX)
