# Supplementary material for: The Low-Cost Compound Lignosulfonic Acid (LA) Exhibits Broad-Spectrum Anti-HIV and Anti-HSV Activity and Has Potential for Microbicidal Applications
Source: PLoS One. 2015 Jul 1;10(7):e0131219. doi: 10.1371/journal.pone.0131219 (PMC4488490; doi:10.1371/journal.pone.0131219)
Supplement: S1 Fig — The indicated cell surface markers highlight the purity of the MDDC population used in our experiments. The white histograms show the background fluorescence. The filled grey histograms indicate the expression levels of six cell surface markers, specific for MDDCs after treatment with the growth factors IL-4 and GM-CSF. (DOCX) [file pone.0131219.s001.docx]

*Supporting Information PLoS ONE (Gordts SC et al.)*

**The Low-cost Compound Lignosulfonic Acid (LA) Exhibits Broad-spectrum Anti-HIV and Anti-HSV Activity and has Potential for Microbicidal Applications.**

***Short title: Dual Anti-HIV and Anti-HSV Activity of LA.***

Stephanie C. Gordts ^1#^, Geoffrey Férir ^1#^, Thomas D’huys^1^, Mariya I. Petrova^2,3^, Sarah Lebeer^2,3^, Robert Snoeck^1^, Graciela Andrei^1^, Dominique Schols^1^*.

**Supporting Information**

**S1 Fig. Specific cell markers for monocyte-derived dendritic cells (MDDCs).** The indicated cell surface markers highlight the purity of the MDDC population used in our experiments. The white histograms show the background fluorescence. The filled grey histograms indicate the expression levels of six cell surface markers, specific for MDDCs after treatment with the growth factors IL-4 and GM-CSF.
