# Supplementary material for: The Low-Cost Compound Lignosulfonic Acid (LA) Exhibits Broad-Spectrum Anti-HIV and Anti-HSV Activity and Has Potential for Microbicidal Applications
Source: PLoS One. 2015 Jul 1;10(7):e0131219. doi: 10.1371/journal.pone.0131219 (PMC4488490; doi:10.1371/journal.pone.0131219)
Supplement: S2 Fig — SupT1 cells were incubated with a high amount of HIV-1 NL4.3 virus (positive untreated control) and combined with or without compounds for 2 hours at RT. Thereafter, cells were extensively washed and gp120 binding was evaluated in all the virus treated conditions with the anti-human 2G12 mAb + RaH-IgG-FITC. The bars represent the percentages of anti-gp120 binding relative to the positive control (d). Each value represents the mean ± SEM of 3 independent experiments. * p<0.05, ** p<0.01, *** p<0.005, *** p<0.001 compared to the nontreated control, according to the one-way Anova and Dunnett’s multi comparison post-hoc test. (DOCX) [file pone.0131219.s002.docx]

*Supporting Information PLoS ONE (Gordts SC et al.)*

**The Low-cost Compound Lignosulfonic Acid (LA) Exhibits Broad-spectrum Anti-HIV and Anti-HSV Activity and has Potential for Microbicidal Applications.**

***Short title: Dual Anti-HIV and Anti-HSV Activity of LA.***

Stephanie C. Gordts ^1#^, Geoffrey Férir ^1#^, Thomas D’huys^1^, Mariya I. Petrova^2,3^, Sarah Lebeer^2,3^, Robert Snoeck^1^, Graciela Andrei^1^, Dominique Schols^1^*.

**Supporting Information**

**Fig S2.** **HIV-1 binding to the cell surface of CD4^+^ T-cells in the presence of LA.** SupT1 cells were incubated with a high amount of HIV-1 NL4.3 virus (positive untreated control) and combined with or without compounds for 2 hours at RT. Thereafter, cells were extensively washed and gp120 binding was evaluated in all the virus treated conditions with the anti-human 2G12 mAb + RaH-IgG-FITC. The bars represent the percentages of anti-gp120 binding relative to the positive control (d). Each value represents the mean ± SEM of 3 independent experiments. * p<0.05, ** p<0.01, *** p<0.005, *** p<0.001 compared to the nontreated control, according to the one-way Anova and Dunnett’s multi comparison post-hoc test.
