# Supplementary material for: The Low-Cost Compound Lignosulfonic Acid (LA) Exhibits Broad-Spectrum Anti-HIV and Anti-HSV Activity and Has Potential for Microbicidal Applications
Source: PLoS One. 2015 Jul 1;10(7):e0131219. doi: 10.1371/journal.pone.0131219 (PMC4488490; doi:10.1371/journal.pone.0131219)
Supplement: S1 Table — (DOCX) [file pone.0131219.s003.docx]

*Supporting Information PLoS ONE (Gordts SC et al.)*

**The Low-cost Compound Lignosulfonic Acid (LA) Exhibits Broad-spectrum Anti-HIV and Anti-HSV Activity and has Potential for Microbicidal Applications.**

***Short title: Dual Anti-HIV and Anti-HSV Activity of LA.***

Stephanie C. Gordts ^1#^, Geoffrey Férir ^1#^, Thomas D’huys^1^, Mariya I. Petrova^2,3^, Sarah Lebeer^2,3^, Robert Snoeck^1^, Graciela Andrei^1^, Dominique Schols^1^*.

**Supporting Information**

**S1 Table. *Lactobacillus* strains used in this study.**

| **Strain** | **Relevant genotype/description** | **reference/source** |
| --- | --- | --- |
| **Gastrointestinal *Lactobacillus* strains** | | |
| *L. rhamnosus* GG (ATCC 53103) | Wild-type, fecal isolate | [1] |
| **Vaginal *Lactobacillus* strains** | | |
| *L. rhamnosus* GR-1 ATCC 55826 | Wild-type, female urethra isolate | [2,3], ATCC |
| *L. reuteri* RC-14  ATCC 55845 | Wild-type, female vaginal isolate | [4,5], ATCC |
| *L. crispatus*  NCIMB 4505 | Wild-type, human vaginal isolate | [6], LMG/BCCM |
| *L. jensenii*  ATCC 25258 | Wild-type, human vaginal isolate | [7], LMG/BCCM |
| *L. gasseri*  ATCC 33323 | Wild-type, human vaginal isolate | [8], LMG/BCCM |
| *L. helveticus*  LG0301 | Wild-type, human vaginal isolate | Mario Van Eechoutte Laboratory Bacteriology Research, UGent |
| *L. helveticus*  LG0373 | Wild-type, human vaginal isolate | Mario Van Eechoutte Laboratory Bacteriology Research, UGent |
| *L. plantarum*  CMPG5300 | Wild-type, human vaginal isolate | [9] |

**References S1 Table**

1. Kankainen M, Paulin L, Tynkkynen S, von Ossowski I, Reunanen J, et al. (2009) Comparative genomic analysis of Lactobacillus rhamnosus GG reveals pili containing a human- mucus binding protein. Proc Natl Acad Sci U S A 106: 17193-17198.

2. Chan RC, Reid G, Bruce AW, Costerton JW (1984) Microbial colonization of human ileal conduits. Appl Environ Microbiol 48: 1159-1165.

3. Chan RC, Reid G, Irvin RT, Bruce AW, Costerton JW (1985) Competitive exclusion of uropathogens from human uroepithelial cells by Lactobacillus whole cells and cell wall fragments. Infect Immun 47: 84-89.

4. Reid G (1999) The scientific basis for probiotic strains of Lactobacillus. Appl Environ Microbiol 65: 3763-3766.

5. Reid G, Bruce AW, Fraser N, Heinemann C, Owen J, et al. (2001) Oral probiotics can resolve urogenital infections. FEMS Immunol Med Microbiol 30: 49-52.

6. Moore WE, Hash DE, Holdeman LV, Cato EP (1980) Polyacrylamide slab gel electrophoresis of soluble proteins for studies of bacterial floras. Appl Environ Microbiol 39: 900-907.

7. Gasser F, Mandel M, Rogosa M (1970) Lactobacillus jensenii sp.nov., a new representative of the subgenus Thermobacterium. J Gen Microbiol 62: 219-222.

8. Lauer E, Kandler O (1980) Lactobacillus gasseri sp. nov., a new species of the subgenus Thermobacterium. Zentbl Bakteriol Mikrobiol 75-78.

9. Malik S, Petrova MI, Claes IJ, Verhoeven TL, Busschaert P, et al. (2013) The highly autoaggregative and adhesive phenotype of the vaginal Lactobacillus plantarum strain CMPG5300 is sortase dependent. Appl Environ Microbiol 79: 4576-4585.
