# Supplementary material for: The Low-Cost Compound Lignosulfonic Acid (LA) Exhibits Broad-Spectrum Anti-HIV and Anti-HSV Activity and Has Potential for Microbicidal Applications
Source: PLoS One. 2015 Jul 1;10(7):e0131219. doi: 10.1371/journal.pone.0131219 (PMC4488490; doi:10.1371/journal.pone.0131219)
Supplement: S2 Table — (DOCX) [file pone.0131219.s004.docx]

*Supporting Information PLoS ONE (Gordts SC et al.)*

**The Low-cost Compound Lignosulfonic Acid (LA) Exhibits Broad-spectrum Anti-HIV and Anti-HSV Activity and has Potential for Microbicidal Applications.**

***Short title: Dual Anti-HIV and Anti-HSV Activity of LA.***

Stephanie C. Gordts ^1#^, Geoffrey Férir ^1#^, Thomas D’huys^1^, Mariya I. Petrova^2,3^, Sarah Lebeer^2,3^, Robert Snoeck^1^, Graciela Andrei^1^, Dominique Schols^1^

**Supporting Information**

**S2 Table.** Virus inactivation of the laboratory-adapted NL4.3 strain in MT-4 cells.

| product | **200 pg virus/well** | | **100 pg virus/well** | | **20 pg virus/well** | |
| --- | --- | --- | --- | --- | --- | --- |
|  | concentration product/well | % block | concentration product/well | % block | concentration product/well | % block |
| **LA low** | 200 ng/ml | 0 | 100 ng/ml | 4 | 20 ng/ml | 41 |
|  | 40 ng/ml | 1 | 20 ng/ml | 2 | 4 ng/ml | 51 |
|  | 8 ng/ml | 1 | 4 ng/ml | 0 | 0.8 ng/ml | 26 |
| **PRO-2000** | 200 ng/ml | 7 | 100 ng/ml | 18 | 20 ng/ml | 82 |
|  | 40 ng/ml | 2 | 20 ng/ml | 6 | 4 ng/ml | 40 |
|  | 8 ng/ml | 0 | 4 ng/ml | 0 | 0.8 ng/ml | 26 |
| **HHA** | 200 ng/ml | 34 | 100 ng/ml | 51 | 20 ng/ml | 68 |
|  | 40 ng/ml | 7 | 20 ng/ml | 9 | 4 ng/ml | 37 |
|  | 8 ng/ml | 5 | 4 ng/ml | 1 | 0.8 ng/ml | 7 |
| **Aldrithiol** | 200 ng/ml | 75 | 100 ng/ml | 87 | 20 ng/ml | 96 |
|  | 40 ng/ml | 50 | 20 ng/ml | 71 | 4 ng/ml | 89 |
|  | 8 ng/ml | 2 | 4 ng/ml | 2 | 0.8 ng/ml | 22 |
